# The transcription factor MYC2 positively regulates terpene trilactone biosynthesis through activating *GbGGPPS* expression in *Ginkgo biloba*

**DOI:** 10.1093/hr/uhae228

**Published:** 2024-08-09

**Authors:** Jiarui Zheng, Yongling Liao, Jiabao Ye, Feng Xu, Weiwei Zhang, Xian Zhou, Lina Wang, Xiao He, Zhengyan Cao, Yuwei Yi, Yansheng Xue, Qiangwen Chen, Jiaxing Sun

**Affiliations:** College of Horticulture and Gardening, Yangtze University, Jingzhou 434025, China; College of Horticulture and Gardening, Yangtze University, Jingzhou 434025, China; College of Horticulture and Gardening, Yangtze University, Jingzhou 434025, China; College of Horticulture and Gardening, Yangtze University, Jingzhou 434025, China; College of Horticulture and Gardening, Yangtze University, Jingzhou 434025, China; College of Horticulture and Gardening, Yangtze University, Jingzhou 434025, China; College of Horticulture and Gardening, Yangtze University, Jingzhou 434025, China; College of Horticulture and Gardening, Yangtze University, Jingzhou 434025, China; College of Horticulture and Gardening, Yangtze University, Jingzhou 434025, China

## Abstract

Terpene trilactones (TTLs) have important medicinal value, but their low content in *Ginkgo biloba* leaves makes their exploitation extremely costly, thereby limiting the development of TTL-related industries. It was found that exogenous methyl jasmonate (MeJA) treatment increased the accumulation of TTLs, but the molecular mechanism is still unclear. Here, we identified two bHLH transcription factors in *G. biloba*, with the protein subcellular localizations in the nucleus. Expression of GbMYC2s was strongly induced by MeJA treatment, and the interactions between GbJAZs and GbMYC2s were demonstrated by yeast two-hybrid and bimolecular fluorescence complementation experiments. Overexpression of *GbMYC2_4* and *GbMYC2_5* enhanced *Arabidopsis* root sensitivity and significantly increased TTL content. In addition, *GbGGPPS* was found to be a common target of GbMYC2_4 and GbMYC2_5 by yeast one-hybrid, electrophoretic mobility shift, and dual-luciferase reporter assays and DAP-seq, and they achieved regulation of *GbGGPPS* by binding to the G-box. Further findings revealed that GbMYC2_4 and GbMYC2_5 bind the G-box not universally but selectively. Our study revealed that jasmonic acid signaling mediates TTL biosynthesis through the GbJAZ–GbMYC2–*GbGGPPS* module, which enriches the terpenoid biosynthesis regulatory networks and provides a research basis and target genes for enhancing TTL content through genetic engineering.

## Introduction

Terpene trilactones (TTLs) are natural products that are unique to *Ginkgo biloba*, and include ginkgolide and bilobalide [1–3]. TTLs are platelet-activating factor receptor antagonists with anti-inflammatory and neuroprotective effects that have important medicinal value and application prospects in clinical practice [4–7], and have therefore received increasing research interest. To date, *G. biloba* leaves are the main source of TTLs, which cannot yet be synthesized industrially. However, the content of TTLs in ginkgo is ~6% (3.1% ginkgolide and 2.9% bilobalide) of leaf extract [8]. This does not adequately meet clinical and production needs, thereby seriously restricting the application and industrial development of TTLs. Therefore, understanding the regulation of TTL biosynthesis is needed to provide new strategies for the efficient production of TTLs and to support the development of the TTL industry.

Due to the high medicinal value of TTLs, many studies have focused on elucidating the synthetic pathways of TTLs. The synthesis of TTLs in ginkgo is catalyzed by specific enzymes, and these enzymes are encoded by genes that have been identified [2, 9]. The precursors of TTL biosynthesis are isopentyl diphosphate (IPP) and dimethylallyl diphosphate (DMAPP), which are synthesized via the mevalonate (MVA) pathway in the cytoplasm and the methyl-d-erythritol phosphate (MEP) pathway in the plastid [10]. Subsequently, ginkgolide and bilobalide were synthesized in *G. biloba* with the catalysis of farnesyl pyrophosphate synthase (FPPS), geranylgeranyl diphosphate synthase (GGPPS), levopimaradiene synthase (LPS), and cytochrome P450 monooxygenase (CYP) [11, 12]. Geranylgeranyl diphosphate synthase works at the branching point of isoprenoid synthesis and catalyzes IPP and DMAPP to generate C-20 geranylgeranyl diphosphate, which is the rate-limiting step of TTL synthesis [11, 12]. Studies in other plants have also shown that the overexpression of *GGPPS* enhances terpene synthesis [13, 14]. Given the important role of GGPPS in TTL biosynthesis, it could be a candidate target gene.

Jasmonic acid (JA) has a positive regulatory role in the synthesis of plant secondary metabolites, and JA signaling is often accomplished through MYC2. JA signaling is commonly defined as COI1/JAZs/MYC2 [15, 16]. MYC2 is involved in plant secondary metabolic processes as an essential regulator in JA signaling. The bHLH family is a large family of transcription factors (TFs) with 85 members in *G. biloba*, and is divided into 17 subfamilies [17]. GbMYC2 belongs to III(d + e) and can bind to the G-box (CACGTG) motif [18, 19], and its regulatory role in the synthesis of plant secondary metabolites has been widely reported. In *Arabidopsis thaliana*, AtMYC2 directly binds to promoters to activate *TPS21* and *TPS11* expression and enhance the synthesis of sesquiterpenes [20]. In *Nicotiana tabacum*, NtMYC2 promotes the transcription of *PMT*, a key enzyme gene in the nicotine biosynthesis pathway, to enhance nicotine content [21]. In *Freesia hybrida*, FhMYC2 regulates linalool biosynthesis by regulating *FhTPS1* [22]. In *Tripterygium wilfordii*, TwMYC2a and TwMYC2b negatively regulate triptolide biosynthesis [23]. In addition, MYC2 is involved in the synthesis of terpenoids in *Catharanthus roseus* [24], *Salvia miltiorrhiza* [25], and *Taxus chinensis* [26]. Previous studies have shown that methyl jasmonate (MeJA) treatment increases the TTL content in ginkgo leaves [3], but the regulatory mechanisms involved are not understood. Therefore, it is improtant to elucidate whether MYC2 TFs are regulating TTL biosynthesis.

Here, we identified GbMYC2 transcription factors GbMYC2_4 and GbMYC2_5 localized in the nucleus, which are able to promote TTL biosynthesis and accumulation. Yeast two-hybrid (Y2H) and bimolecular fluorescence complementation (BiFC) assays showed that GbMYC2_4 and GbMYC2_5 are able to intercalate with GbJAZs. The TTL biosynthesis key gene, *GbGGPPS*, is a common target of GbMYC2_4 and GbMYC2_5. Y1H assay, electrophoretic mobility shift assay (EMSA), dual-luciferase assay, and DAP-seq analyses showed that GbMYC2_4 was able to selectively bind directly to the G-box motif in the *GbGGPPS* promoter, and GbMYC2_5 prefers to bind neighboring series A/T-enriched G-box motifs and activate *GbGGPPS* expression. Furthermore, a molecular model of JA signaling-mediated GbMYC2 regulation of TTL synthesis was drawn. These results enrich the TTL biosynthesis regulatory network, which is important for an in-depth understanding of TTL synthesis and the enhancement of TTL content through genetic engineering.

## Results

### Sequence structure, functions, and subcellular localization analysis of GbMYC2_4 and GbMYC2_5

**Figure 1:**
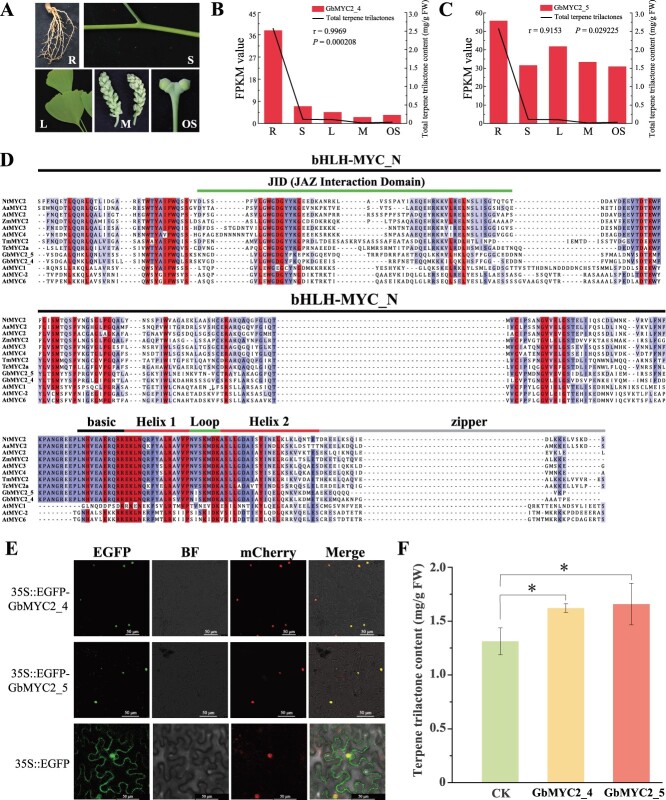
GbMYC2_4 and GbMYC2_5 participate in TTL biosynthesis. **A** Different tissues of 34-year-old *Ginkgo biloba*. R, root; S, stem; L, leaf; M, microstrobilus; OS, ovulate strobilus. **B**, **C** Expression levels of GbMYC2_4 (**B**) and GbMYC2_5 (**C**) were strongly correlated with TTL content in different tissues of *G. biloba*. **D** Gene structures of GbMYC2_4 and GbMYC2_5 and alignment of their conserved domains. Different shades of color indicate different comparison similarities. The conserved bHLH–MYC and helix–loop–helix (HLH) domains are marked above the structures. GbMYC2_4 and GbMYC2_5 amino acid sequences were translated from genome sequences, and other amino acid sequences were retrieved from GenBank. **E** Subcellular localization analyses of GbMYC2_4 and GbMYC2_5 in *N. benthamiana*. **F** Transient overexpression of GbMYC2_4 and GbMYC2_5 increased the content of TTLs in ginkgo leaves. Group settings: C (control) versus M4 (overexpression of GbMYC2_4), CK (control) versus M5 (overexpression of GbMYC2_5). Error bars indicate the standard deviation of three biological replicates. Asterisks indicate significant differences. ^*^P < 0.05.

An analysis of previous studies revealed that GbMYC2_4 and GbMYC2_5 were specifically expressed in different tissues of *G. biloba* (Fig. 1A) and strongly correlated with TTL content ([2]; Fig. 1B and C). The two full-length open reading frames (ORFs), named GbMYC2_4, and GbMYC2_5, were isolated using specific primers (Supplementary Data Table S1). The ORF of GbMYC2_4 contained 1893 bp and encoded a protein of 631 amino acids. The ORF of GbMYC2_5 contained 1758 bp and encoded a protein of 586 amino acids. The results of Protein BLAST and the sequence comparison showed that GbMYC2_4 and GbMYC2_5 have a highly conserved bHLH–MYC domain in the N-terminal region and an HLH DNA-binding domain in the C-terminal region (Fig. 1D). They showed a highly conservative amino acid sequence with other MYC2s, including *Taxus mairei* [26], *Taxus chinensis* [26], *A. thaliana* [27], *N. tabacum* [21], *Zea mays* [28], and *Artemisia annua* [29], while they showed poor sequence similarity with AtMYC1, AtMYC-2 (EGL3, belongs to the bHLH family) and AtMYC6.

To investigate the subcellular localization of GbMYC2_4 and GbMYC2_5, we transiently expressed SubN*-CaMV35S::EGFP-GbMYC2_4/GbMYC2_5* (with p2300-*CaMV35S::H2B-mCherry*, 1:1), and SubN*-CaMV35S::EGFP* (control vector) in *Nicotiana benthamiana* leaves, respectively. Confocal laser scanning microscope observations revealed that the control vector GFP signal was distributed in the cell membrane and nucleus (Fig. 1E). In contrast, the GFP signal of EGFP-GbMYC2_4/GbMYC2_5 was detected only in the nucleus and co-localized with the H2B-mCherry signal (Fig. 1E), indicating that both GbMYC2_4 and GbMYC2_5 are located in the nucleus.

### GbMYC2_4 and 5 positively regulate terpene trilactone biosynthesis

To fully clarify the function of GbMYC2_4 and 5 in TTL accumulation, they were transiently overexpressed in *Ginkgo* leaves. The TTL content in leaves overexpressing GbMYC2_4 was 1.6221 mg/g FW, which was significantly higher than the TTL content [control group, empty vector (CK), 1.3128 mg/g FW] in leaves injected with the empty vector (Fig. 1F). The TTL content in SK-GbMYC2_5-overexpressing leaves was 1.6581 mg/g FW, which was significantly higher than in CK (Fig. 1F). These results indicate that GbMYC2_4 and GbMYC2_5 were able to promote the accumulation of TTLs in *Ginkgo* leaves.

### Methyl jasmonate induces the accumulation of terpene trilactones and the upregulation of GbMYC2s

**Figure 2:**
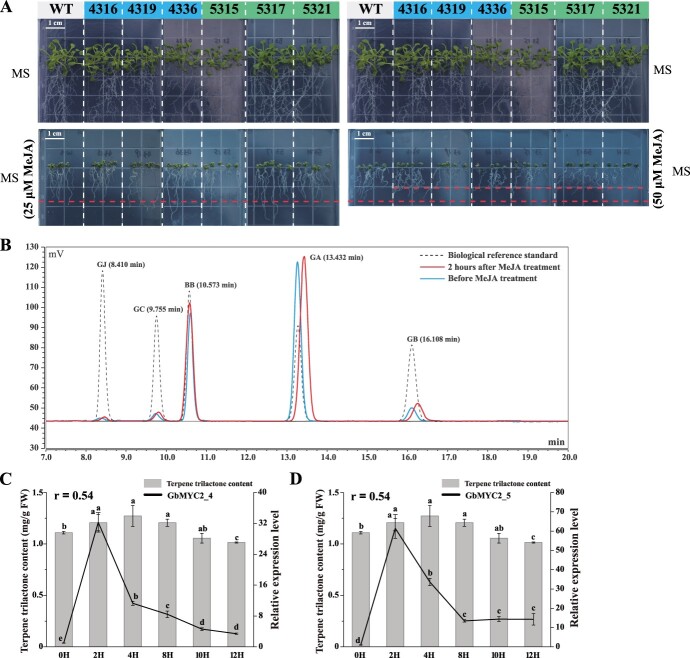
MeJA inhibited root elongation in *Arabidopsis* overexpressing GbMYC2 and increased TTL accumulation and GbMYC2 expression. **A** Root-length sensitivity analysis showed that GbMYC2_4 and GbMYC2_5 were highly sensitive to MeJA. **B** Chromatograms of the samples and biological reference standards (dotted lines). The time range is 7–20 min. Red lines represent samples induced by MeJA for 2 h. Green lines represent samples before MeJA induction. GJ, ginkgolide J; GC, ginkgolide C; BB, bilobalide; GA, ginkgolide A; GB, ginkgolide B. **C**, **D** Correlation analysis of GbMYC2_4 (**C**) and GbMYC2_5 (**D**) expression levels and TTL content in leaves of *G. biloba* at different time points after 10 mM MeJA treatment. *GbGAPDH* was used as an internal control. Data represent the means ± standard deviation of three replicates. Different lower-case letters indicate significant differences at the *P* < 0.05 level.

When grown on MS medium, both wild-type and overexpression lines grew healthily. However, when 25 μM MeJA was added, the root length of *Arabidopsis* lines overexpressing GbMYC2 was suppressed as well as that of the wild type, but the root lengths of wild-type and overexpression plants were basically the same (Fig. 2A). When the MeJA concentration was raised to 50 μM, the root lengths of wild-type *Arabidopsis* lines were similar to those grown on MS medium with 25 μM MeJA, but the primary root growth of the transgenic lines was more strongly inhibited, especially for lines 4319, 5319, 5317, and 5321, which showed stronger sensitivity to MeJA (Fig. 2A). The TTL contents in the *G. biloba* plants were determined using high-performance liquid chromatography–evaporative light-scattering detection (HPLC–ELSD). The chromatographic peaks of the *G. biloba* sample and standard are shown in Fig. 2B. MeJA treatment was able to induce TTL accumulation and reach the maximum content at 4 h, followed by a gradual decrease (Fig. 2B). Compared with the 0 h (1109.6 μg/g FW), the TTL content was increased by 8.7–14.6% (1206.4–1271.9 μg/g FW) after MeJA induction.

To confirm whether GbMYC2 responded to MeJA, the GbMYC2_4 and GbMYC2_5 expression levels after MeJA treatment were analyzed using qRT–PCR at different time points. The results showed that MeJA was effective in inducing GbMYC2_4 and GbMYC2_5 expression. The transcript levels of GbMYC2_4 and GbMYC2_5 increased rapidly after 2 h of MeJA treatment, peaked at 2 h, and then slowly declined until 12 h (Fig. 2C and D). Correlation analysis showed that the expression levels of GbMYC2_4 (Fig. 2C, *r* = 0.54, *P* < 0.05) and GbMYC2_5 (Fig. 2D, *r* = 0.54, *P* < 0.05) were highly correlated with TTL content.

### GbMYC2 interacts with GbJAZ proteins

**Figure 3:**
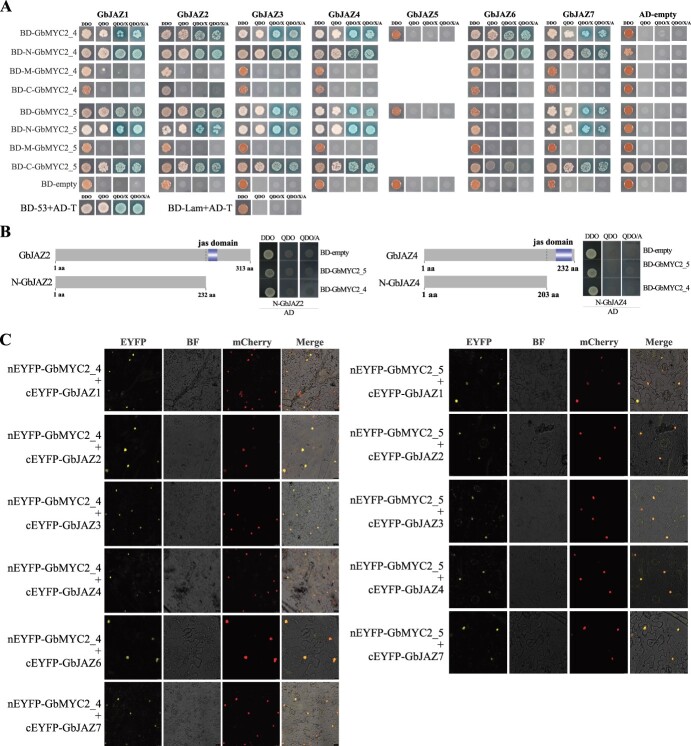
GbMYC2_4 and GbMYC2_5 transcription factors interact with GbJAZ1/2/3/4/5/6/7 in yeast cells. GbMYC2_4 does not interact with GbJAZ5 and GbMYC2_5 does not interact with GbJAZ5/6. **A** GbMYC2 proteins interact with GbJAZs. Y2H yeast cells with bait vector and Y187 yeast cells with prey vector were mixed and cultured for 12 h, and were grown on SD/−Trp/−Leu (DDO), SD/−Trp/−Leu/−His/−Ade (QDO), QDO/X, and QDO/X/A medium to test the interaction. pGBKT7–53 and pGADT7-T were used as positive controls, and pGBKT7-lam and pGADT7-T were used as negative controls. **B** GbMYC2_4 and GbMYC2_5 did not interact with the N-termini of GbJAZ2 and GbJAZ4 in Y2H assays. **C** BiFC assay to detect interactions of GbMYC2 [fused with the C-terminal fragment of yellow fluorescent protein (YFP) in pNC-BiFC-Enn] with GbJAZs (fused with the N-terminal fragment of YFP in vector pNC-BiFC-Ecn). Two *Agrobacterium* GV3101-containing recombinant vectors were mixed and co-infiltrated into leaves of *N. benthamiana*. YFP fluorescence was detected 3 days after infiltration. Scale bars = 50 μm.

To determine whether GbMYC2_4 and GbMYC2_5 interact with JAZ proteins, we cloned seven *GbJAZ*s from the *G. biloba* cDNA library. First, qRT–PCR analysis showed that, except for JAZ5, the six JAZs exhibited basically the same trend of changes induced by MeJA (Supplementary Data Fig. S1). Further correlation analysis showed that the expression changes of JAZ5 were opposite to those of GbMYC2_4 and GbMYC2_5 (Supplementary Data Fig. S1C). Next, autoactivation of two MYC2s and their fragments (N, M, C-GbMYC2) was performed with BD-empty as a control. The autoactivation test showed that GbMYC2_4, GbMYC2_5 and their truncated fragment (N, M, C-GbMYC2) baits did not autonomously activate the reporter genes in Y2HGold strains in the absence of a prey protein (Supplementary Data Fig. S2). Therefore, GbMYC2_4 and 5 and their truncated fragments were used in subsequent experiments. Finally, Y2H assays were performed. The Y2H assays indicated that GbMYC2_4 and N-GbMYC2_4 could interact with GbJAZ1/2/3/4/6/7 proteins. GbMYC2_5, N-GbMYC2_5, and C-GbMYC2_5 could interact with GbJAZ1/2/3/4/7 proteins in Y2H yeast cells (Fig. 3A). Neither GbMYC2_4 nor GbMYC2_5 can interact with JAZ5, and GbMYC2_5 does not interact with GbJAZ6.

**Figure 4:**
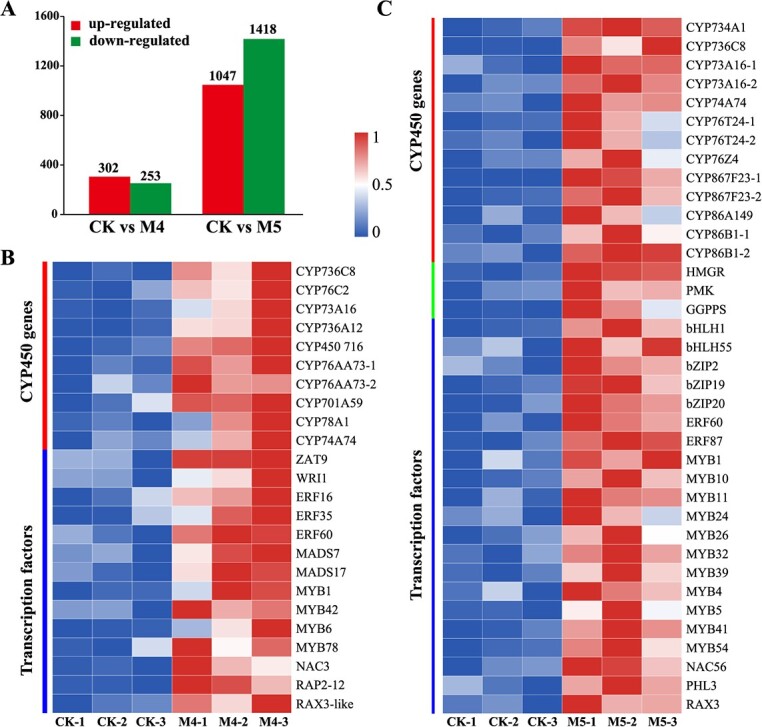
Transcriptome analysis shows upregulated genes in M4 (overexpression of GbMYC2_4) and M5 (overexpression of GbMYC2_5). **A** Distribution of upregulated or downregulated genes by M4 and M5 in RNA-seq experiment. **B**, **C** Heat maps of representative upregulated genes induced by M4 and M5.

To explore the specificity of the interactions between GbJAZs and MYC2s, N-JAZ2 and N-JAZ4 fragments with the Jas region removed were used for Y2H experiments. As shown in Fig. 3B, neither N-JAZ2 nor N-JAZ4 could interact with GbMYC2_4 and GbMYC2_5.

Furthermore, a BiFC assay was performed to examine the GbMYC2-GbJAZ interactions in tobacco plants. When nEYFP-GbMYC2_4 was co-expressed with cEYFP-GbJAZ1/2/3/4/6/7, respectively, in wild *N. benthamiana* leaves, strong yellow YFP fluorescence was detected in the nucleus of the epidermal cells of leaves and co-localized with the mCherry signal of H2B-mCherry (Fig. 3C). As shown in Fig. 3C, strong fluorescence was observed in the nucleus of *N. benthamiana* cells after co-expression of nEYFP-GbMYC2_5 and cEYFP-GbJAZ1/2/3/4/7. These results further confirm the interaction of GbMYC2_4 and 5 with GbJAZs.

### Transcriptome profiling of MYC2-regulated genes

To further identify TTL biosynthetic enzyme genes that may be regulated by GbMYC2_4 and GbMYC2_5, three biological replicates of CK (control group, empty vector), M4 (overexpression of GbMYC2_4), and M5 (overexpression of GbMYC2_5) were used for transcriptome sequencing. Considering the direct role of *CYP* genes in TTL biosynthesis [11], differentially expressed *CYP* genes were identified. Rigorous statistical analysis was performed on M4, M5, and CK transcriptome data (Supplementary Data Table S2). We identified 1047 upregulated and 1418 downregulated genes from CK versus M4 (Fig. 4A), of which 10 *CYP* genes were upregulated (Fig. 4B). In the CK versus M5 comparison group, we identified a total of 302 upregulated genes and 253 downregulated genes (Fig. 4A), of which 13 *CYP* genes, one *HMGR*, one *PMK*, and one *GGPPS* were upregulated (Fig. 4C). In addition, we identified 14 and 21 TFs upregulated by MYC2 from CK versus M4 and CK versus M5, respectively (Fig. 4B and C). These TFs belong to the MYB, ERF, MADS, NAC, bZIP, and bHLH families, which may be involved in regulating TTL biosynthesis.

KEGG enrichment analysis showed that 26 and 37 pathways (Q-value ≤0.05) were significantly enriched in CK versus M4 and CK versus M5 comparisons, respectively (Supplementary Data Table S3). It was observed that a total of eight pathways were significantly enriched in both groups. Among these eight pathways, six were related to metabolism, namely phenylpropanoid biosynthesis, starch and sucrose metabolism, cyanoamino acid metabolism, flavonoid biosynthesis, stilbenoid, diarylheptanoid and gingerol biosynthesis, and tryptophan metabolism. Furthermore, in addition to monoterpenoid biosynthesis, ubiquinone and other terpenoid-quinone biosyntheses being associated with terpenoid anabolism, several other primary metabolic mechanisms, such as the MAPK signaling pathway, glycine, serine and threonine metabolism, glycerophospholipid metabolism, and zeatin biosynthesis, were also significantly enriched in the CK versus M4 comparison. In the CK versus M5 comparison, three were related to the metabolism of terpenoids, namely carotenoid biosynthesis, diterpenoid biosynthesis, and indole alkaloid biosynthesis; and several other primary metabolic mechanisms, such as photosynthesis, MAPK signaling pathway, DNA replication, amino sugar and nucleotide sugar metabolism, fructose and mannose metabolism, and the circadian rhythm, were also significantly enriched.

### Identification of *GGPPS* as a target gene of GbMYC2_4 by DAP-seq

**Figure 5:**
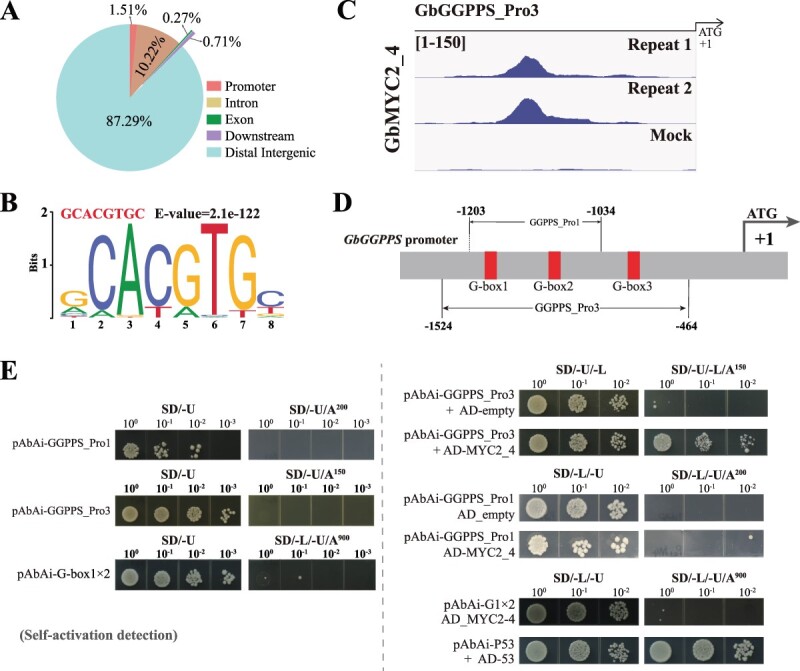
Genome-wide identification of GbMYC2_4 binding sites and motifs. **A** Genome-wide distribution analysis of GbMYC2_4 binding peaks. **B** A GCACGTGC core motif identified by MEME-ChIP was found to be bound by GbMYC2_4. **C** IGV browser view of GbMYC2_4 binding on its target genes *GbGGPPS*. GbMYC2_4-binding peaks (repeats 1 and 2) and negative control (mock) in the promoters of *GbGGPPS* by DAP-seq. ‘[1 to 150]’ shows the scale bar for binding peak heights. The translational start site (ATG) is shown at position +1. **D** Schematic diagram of the promoters (GbGGPPS_Pro3) of the *GbGGPPS* genes. The translational start site (ATG) is shown at position +1. **E** Y1H verified that GbMYC2_4 can bind to GbGGPPS_Pro3 but not to GbGGPPS_Pro1 and G1 × 2. Y1HGold containing GbGGPPS_Pro3 and pGADT7-GbMYC2_4 was able to grow on screening medium containing 150 ng ml^−1^ aureobasidin A. The empty vector pGADT7 was used as a negative control. Yeast cultures were diluted 10-fold and spotted on the medium.

In order to find the possible downstream target genes of GbMYC2_4, we used GbMYC2_4 as a bait to select the DAP-seq method to identify the downstream target genes [30, 31]. We identified 72 763 and 66 045 peaks from the two replicates, respectively, of which 44 565 peaks were shared (Supplementary Data Fig. S3A). GbMYC2_4 binding sites were concentrated near transcription start sites and distributed mainly in distal intergenic and intron regions (Fig. 5A). Enrichment analysis revealed that the G-box motif (GCACGTGC) was one of the motifs with the highest enrichment of GbMYC2_4 binding peaks (Fig. 5B). In addition to the G-box, we determined that GbMYC2_4 binds five other unidentified motifs, with the sequences AMCATATGBT, CCAAYGCCCCWYTTAGGGGTT, TRTGCACAAAA, CACATTWAACACATG, and AAATRGGGYRCATRGAA (Supplementary Data Fig. S3B–G). In the DAP-seq data, we found that the *GbGGPPS* promoter, the target of GbMYC2_5, is also a candidate target gene of GbMYC2_4 (Supplementary Data Table S4). In agreement, we detected strong GbMYC2_4-binding peaks in the promoter region of *GbGGPPS* (Fig. 5C), and this peak annotated to the promoter sequence for GGPPS_Pro3. Y1H assay results show that GbMYC2_4 was able to bind to GbGGPPS_Pro3 (Fig. 5D). Further analysis revealed that GbGGPPS_Pro3 contains GbGGPPS_Pro1; however, GbMYC2_4 is not able to bind to GbGGPPS_Pro1 (Fig. 5E).

### GbMYC2_5 positively regulates *GbGGPPS* expression

**Figure 6:**
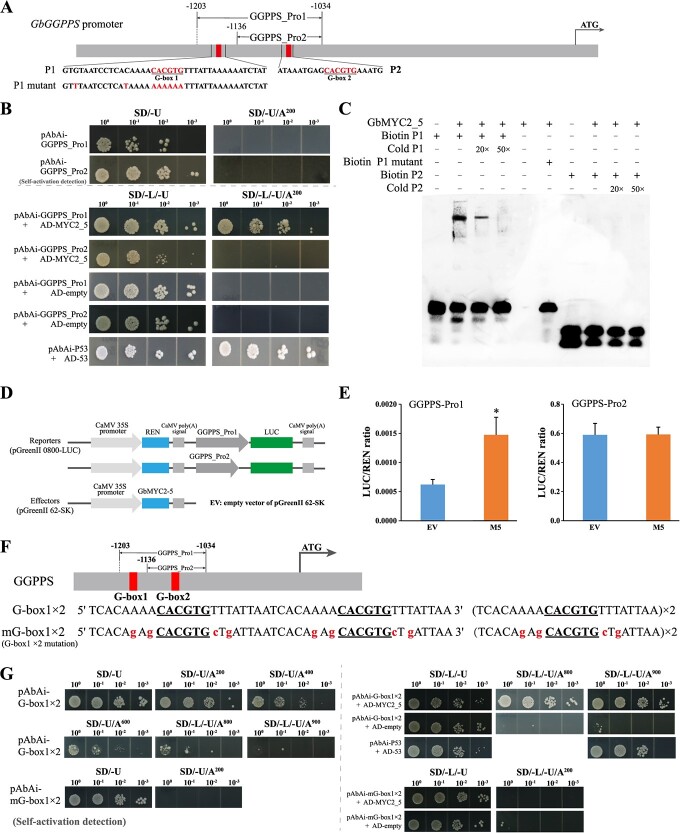
GbMYC2_5 can specifically bind to the G-box to mediate *GbGGPPS* expression in *G. biloba*. **A** Schematic diagrams of the *GbGGPPS* promoter showing potential MYC2-binding sites. GGPPS_Pro1 and GGPPS_Pro2 was used in Y1H and transactivation assays. GGPPS_Pro1 and GGPPS_Pro2 containing two and one putative GbMYC2_5 binding sites, respectively, are indicated with vertical red bars. P1 and P2 have sequences centered on G-box motifs, and G-box elements are marked by underlining. **B** Y1H assay was used to detect the binding of GbMYC2_5 to GGPPS_Pro1 and GGPPS_Pro2. The negative control was the empty vector pGADT7. Yeast cells were diluted 10-fold and spotted on medium. **C** EMSA showing that GbMYC2_5 binds the P1 (G-box1) motif of the *GbGGPPS* promoter *in vitro* but fails to bind to P2 (G-box2). **D**, **E** In *N. benthamiana*, GbMYC2_5 activates GGPPS_Pro1 but not GGPPS_Pro2. Four biological assays were performed. Asterisks indicate significant differences by Student's *t*-test. ^*^P < 0.05. EV, pGreenII 62-SK empty vector. **F** Artificial double repeated G-box motif sequences; the G-box elements are underlined. mG-box1 is a mutant sequence of G-box1. **G** GbMYC2_5 binds to the G-box1 × 2 motif but not to the mutant mG-box motif. Y1HGold yeast containing G-box1 × 2 could not grow at a concentration of 800 or 900 ng l^−1^ AbA, and addition of GbMYC2_5 allowed Y1HGold yeast to grow on synthetic medium lacking leucine under 800 or 900 ng l^−1^ concentration of AbA.

The transcriptome results showed that *GbMYC2_5* overexpression induced the upregulated expression of *GbGGPPS* (Fig. 4C), and thus *GbGGPPS* may be a potential target of GbMYC2_5. Further analysis showed that the −1203 to −1034 region (GGPPS_Pro1) of the *GbGGPPS* promoter contained two G-boxes (Fig. 6A), and the binding activity of GbMYC2_5 to GGPPS_Pro1 was probed by the yeast one-hybrid (Y1H) assay. The Y1H assay showed that yeast cells transformed with AD-GbMYC2_5 and pAbAi-GbGGPPS_Pro1 grew well on the selected medium, while yeast cells transformed with AD-empty and pAbAi-GbGGPPS_Pro1 did not grow (Fig. 6B). Further, we investigated the binding of GbMYC2_5 to GbGGPPS_Pro2 and found that yeast cells containing both AD-GbMYC2_5 and pAbAi-GbGGPPS_Pro2 plasmids were unable to grow on the selected medium (Fig. 6B). These results indicate that GbMYC2_5 can bind to the G-box1 of *GbGGPPS* promoter (−1203 to −1136 bp region).

MYC2 regulates gene transcription by recognizing and binding to G-box or G-box-like motifs [32, 33], and therefore nine key enzymes genes involved in the TTL biosynthesis pathway, which contain G-box or G-box-like motifs in their promoters, were selected for Y1H assay. Specifically, promoter analysis of structural genes in the TTL synthesis pathway revealed that the promoters of the *LPS*, *AACT1*, *AACT2*, *HMGR1*, *HMGR2*, *HMGS*, *DXS1*, *DXS2*, and *GGPPS2* genes contain one G-box or G-box-like motif (Supplementary Data Fig. S4A). However, the nine promoters were not targets of GbMYC2_5 (Supplementary Data Fig. S4C).

We also further confirmed the binding activity of GbMYC2_5 to the *GbGGPPS* promoter by EMSA assay. We designed P1, P1 mutant, and P2 probes according to the position of the G-box in the *GbGGPPS* promoter (Fig. 6A). We found that GbMYC2_5 protein could bind the P1 sequence, but not the P1 mutant and P2 sequences (Fig. 6C). Consistent with this observation, an excess of unlabeled probes (cold P1, 20× and 50×) competed with the labeled probes for binding to GbMYC2_5 and eliminated migration (Fig. 6C), suggesting that GbMYC2_5 specifically binds the P1 sequence in the *GbGGPPS* promoter. To further determine the activation effect of GbMYC2_5, two firefly LUC reporter constructs driven by GbGGPPS_Pro1 and GbGGPPS_Pro2 were created, while an effector was generated using GbMYC2_5 (Fig. 6D). GbGGPPS_Pro1 has one more promoter fragment containing a G-box than GbGGPPS_Pro2 (Fig. 6A). Transient expression experiments showed that the effector co-expressed with the reporter containing GbGGPPS_Pro1 activated LUC 2.37-fold compared with the control, and the replacement of GbGGPPS_Pro1 with GbGGPPS_Pro2 resulted in a sharp decrease in activity (Fig. 6E). These results indicate that GbMYC2_5 acts as a transcriptional activator of *GbGGPPS* by binding with the G-box, and can specifically select and bind to the G-box.

To explore the reason for the preference of GbMYC2_5 for binding to the G-box1, a careful examination of the *GbGGPPS* promoter revealed that the A/T content of the region near G-box2 (8 bp before and after the extension, 11/16) was lower than that of the region near G-box1 (14/16) (Fig. 6F). Based on the *GbGGPPS* promoter sequence, two artificial repeats of G-box1 (G-box1 × 2) and a mutant (mG-box1 × 2) were designed (Fig. 6F). The results of the Y1H assays showed that GbMYC2_5 binds to G-box1 × 2 but not to the mutant (Fig. 6G). The difference between G-box1 × 2 and mG-box1 × 2 is that the G/C content near the G-box is increased (2/16 to 6/16). Thus, these results suggest that GbMYC2_5 is biased towards binding G-boxes with higher A/T content in nearby regions.

## Discussion

### Methyl jasmonate-mediated GbMYC2 regulates the synthesis of terpene trilactones in *G. biloba*

The biosynthesis of TTLs is tightly regulated and highly coordinated through various metabolic pathways, including the hormonal pathway. Numerous studies have shown that JA is an important regulator of TTL synthesis [3, 34]. However, the exact mechanism by which JA influences TTL synthesis in *G. biloba* is unclear. The MYC2 TFs are considered to be the core regulators of JA signaling [35], and are involved in the accumulation of terpenoids in *A. thaliana* [20], *C. roseus* [24], *T. wilfordii* [23], *T. chinensis* [26], and *S. miltiorrhiza* [36]. In this study, we identified two bHLH TFs of GbMYC2 localized in the nucleus of *G. biloba* (Fig. 1E), which are homologous and have high sequence similarity with AtMYC2, NtMYC2, AaMYC2, ZmMYC7, TmMYC2, and TcMYC2a (Fig. 1D). Transgenic *A. thaliana* overexpressing GbMYC2_4 and GbMYC2_5 was obtained by the floral dip method, and root-length sensitivity analysis showed that GbMYC2_4 and GbMYC2_5 were strongly sensitive to MeJA (Fig. 2A), suggesting that GbMYC2_4 and GbMYC2_5 are key regulatory nodes in JA signaling. In *C. roseus*, CrMYC2 is involved in the synthesis of alkaloids [37]. In *A. annua*, AaMYC2 is involved in the regulation of artemisinin content [29]. In our study, exogenous MeJA treatment was able to increase the expression levels of GbMYC2s, and it is hypothesized that GbMYC2 may have a similar role to CrMYC2 and AaMYC2 after MeJA treatment and is involved in JA signaling. Since ginkgo is a perennial woody plant and it is difficult to obtain stable transgenic strains, a well-established transient overexpression method applied in persimmon [38] and citrus [39, 40] was used to detect the biological function of GbMYC2 in TTL synthesis. The transient expression results showed that *GbMYC2_4* and *GbMYC2_5* overexpression increased the content of TTLs (Fig. 1F). Thus, our study clarifies that GbMYC2 is a positive regulator of ginkgolide synthesis, which is consistent with MYC2 in regulating the synthesis of nicotine [21], paclitaxel [26], artemisinin [29], and linalool [22]. The functional consistency suggests that MYC2 can broadly regulate the synthesis of terpenoids.

### Identification of the key structural domains responsible for the interaction between GbMYC2 and GbJAZs

JA plays an irreplaceable role in the synthesis of secondary metabolites in plants [41, 42]. It has been shown the JAZ protein is a key transcriptional repressor in JA signaling. At low or normal JA concentrations, JAZ can bind to MYC2 to inhibit its activity, and when MeJA treatment or JA concentration is increased, JAZ can be degraded by the JA pathway and release the bound TFs [16, 43–46]. However, the relationship between JAZ and MYC2 in *G. biloba* has not been reported. Earlier studies have shown that the repressor protein JAZ directly interacts with MYC2 in a variety of JA-mediated physiological processes, and thus MYC2 transcription requires JA induction [47–50], which seems to be the reason why MeJA can induce the upregulation of GbMYC2 expression in *G. biloba*. In this study, the expression level of GbMYC2 was significantly increased by MeJA treatment (Fig. 2C and D).

Interaction between JAZ and MYC2 appears to occur universally and across species. A total of 13 JAZ proteins are present in *A. thaliana*, 11 of which can interact with AtMYC2 [18]. In rice, 14 JAZ proteins interact with OsMYC2 [51]. In tomato, MYC2 was able to interact with all 11 JAZ proteins [15]. In maize, ZmMYC2 is able to interact with AtJAZ1 and AtJAZ9 [52]. In this study, the Y2H and BiFC assays showed that GbMYC2_4 was able to interact with six JAZ proteins, and GbMYC2_5 was able to interact with five JAZ proteins (Fig. 3). All these results suggest that JAZ–MYC interactions are universal.

To investigate the region where GbMYC2 interacts with JAZ proteins, GbMYC2_4 and GbMYC2_5 were truncated into three fragments according to the conserved structure domain for the Y2H assay (Supplementary Data Fig. S2). The results showed that N-GbMYC2_4 containing the N-terminal region had the same ability to interact with JAZ proteins as the full-length sequence of GbMYC2_4, while the M-GbMYC2_4 and C-GbMYC2_4 fragments containing the middle region (M) and C-terminal region were unable to interact with JAZ proteins (Fig. 3A). In addition, more surprising results were observed in GbMYC2_5, where the fragments containing the N-terminal or C-terminal region were able to interact with JAZ proteins. As reported, the N-terminal region of MYC2 interacts with JAZ because of the JID domain [18], and our findings confirm this. Also of note is the phenomenon of the C-terminal region of GbMYC2_5 interacting with the JAZ protein. Perhaps EGL3, which is also in the bHLH family in *Arabidopsis*, offers a clue, as the C-terminal region of EGL3 can interact with JAZ1/8 [53]. MYC2 belongs to the bHLH family and also possesses the basic features and functions of bHLH, which might be the reason why C-GbMYC2_5 interacts with JAZ proteins. The Jas motif is the basis for the interaction between JAZ and MYC2 [54]. In our study, JAZ2 and JAZ4 with the Jas structural domain removed were then unable to interact with GbMYC2_4 and GbMYC2_5 (Fig. 3B), and these results suggest that the Jas motif is a key structure in the interaction of JAZ with MYC2. In conclusion, our results suggest that GbMYC2 transcription is regulated by JA signaling and GbJAZ.

### GbMYC2 positively regulates terpene trilactone biosynthesis through transcriptional activation of *GbGGPPS*

Due to the significant medicinal value of TTLs [55, 56], their biosynthetic pathways and regulatory mechanisms have been an important research topic. Thus far, all the genes encoding enzymes involved in TTL biosynthesis have been cloned and identified, including *GGPPS* [57], *LPS* [58], and *CYP450* [59]. In the present study, we found that GbMYC2_5 could bind to the G-box motif in the *GbGGPPS* promoter (Fig. 6), and thus the TTL content was increased when GbMYC2 was overexpressed in *G. biloba* leaves (Fig. 1F). Transient overexpression of *CrGGPPS2* enhanced monoterpene indole alkaloid biosynthesis in *C. roseus* [13], and overexpression of *SmGGPPS* increased the content of tanshinone in the hairy roots of *S. miltiorrhiza* [14]. These studies support our finding that the enhanced expression of *GGPPS* enhances the content of terpenoids.

Previous studies have shown that MYC2 can bind to the G-box (CACGTG) motif and its similar motifs 5′-cacntg-3′, 5′-cacatg-3′, and 5′-(t/c)acgtg-3′1 [19, 32, 33]. In our study, GbMYC2_5 was tested for its ability to bind to 10 promoters containing G-box or G-box-like motifs, and it was found that GbMYC2_5 only binds to *GbGGPPS* promoters containing the G-box, and the remaining nine promoters were not targets of GbMYC2_5 (Fig. 6B, Supplementary Data Fig.S4). Specifically, GbMYC2_5 was able to bind to the GbGGPPS_Pro1 promoter but not to GbGGPPS_Pro2 (Fig. 6B). The results of the dual luciferase assay showed that the LUC activity of GbMYC2_5 bound to the GbGGPPS_Pro1 promoter (containing two G-boxes) was significantly higher than that of the empty vector (EV) group. Furthermore, the LUC activity of GbMYC2_5 bound to the GbGGPPS_Pro2 promoter (containing one G-box) was reduced to the level of EV, indicating that GbMYC2_5 was able to bind to the G-box1 specifically (Fig. 6E). The results of the EMSA assay further indicated that GbMYC2_5 binds to fragments containing G-box1 but not to fragments containing G-box2 (Fig. 6A and C). These results all indicate that GbMYC2_5 specifically binds to G-box1. Meanwhile GbMYC2_4 is able to bind to GbGGPPS_Pro3 (containing G-box1, G-box2, and G-box3) but not to GbGGPPS_Pro1 (containing G-box1 and G-box2), suggesting that GbMYC2_4 is selective for binding to G-box3 (Fig. 5D and E). The main reason for this difference may be related to the distribution of bases in the region near the G-box motif.

### GbMYC2 is biased to bind A/T-enriched G-boxes in nearby regions

Previous studies have shown that MYC2 mainly binds to G-box motifs in promoter sequences to achieve the transcriptional regulation of target genes, with a preference for binding to A/T-rich modules near the G-box motifs [21, 60]. Our results also present similar results showing that increasing the G/C content near G-box1 in the *GGPPS* promoter (2/16 to 6/16) eliminated the binding activity of GbMYC2_5 (Fig. 4F and G). In *T. chinensis*, TcMYC2a can bind to the G-box in the *TASY* promoter [26]. In *Arabidopsis*, pDof2.1 was able to bind to the AtMYC2 promoter sequence containing two E-boxes and specifically bound to the second E-box motif [33]. These studies support our finding that GbMYC2 specifically recognizes and biases binding to a nearby region to A/T-rich G-box motifs in the promoter. Plant secondary metabolite biosynthesis usually involves multiple enzymatic steps, and increasing the production of target metabolites in plants by overexpressing one or two key enzymes is usually inefficient, whereas TFs typically regulate key steps of target metabolite synthesis or even the entire synthetic pathway [29, 61, 62]. Thus, given the critical role of GbMYC2 in JA signaling, as well as its direct regulation of *GbGGPPS* and specific selective binding to G-box motifs, it may become an effective tool for regulating TTL biosynthesis in the future.

### GbMYC2 upregulates multiple *CYP* gene and transcription factor expressions

*CYP* family members have been known to directly catalyze the synthesis of TTLs [11]. We have shown through multiple studies evidence that GbMYC2_5 specifically selects to bind G-box motifs (Fig. 6), and DAP-seq sequencing results have also shown that GbMYC2_4 is able to bind the G-box (Fig. 5). However, analysis of the promoters of those *CYP* genes that are directly involved in the biosynthesis of TTLs did not reveal G-box motifs [11] (Supplementery Data Table S5). Therefore, the four *CYP* genes may not be their targets. From the RNA-seq data, we identified 10 upregulated *CYP* family genes by GbMYC2_4 (Fig. 4B), and further analysis of the 10 *CYP* promoters revealed that 4 promoters (CYP736C8-1, CYP76AA73-1, CYP76AA73-2, and CYP701A59) contained G-box motifs (Supplementery Data Table S6), and we hypothesized that they might be targets of GbMYC2_4. In addition to *GGPPS*, GbMYC2_5-upregulated genes include one HMGR, one PMK, and 13 *CYP* genes. The promoter sequences of CYP736C8-2 and CYP76T24-1 were found to contain G-box motifs, so it is reasonable to hypothesize that CYP736C8 and CYP76T24-1 may be the targets of GbMYC2_5, but this hypothesis needs to be supported by experimental evidence. The MYC2 TF is one of the more widely studied members of the bHLH TFs and has been found to be involved in the regulation of terpenoid biosynthesis genes in many plants [21, 26, 29, 37]. Except for MYC2, terpenoid biosynthesis was also regulated by AP2/ERF, MYB, NAC, WRKY, and bZIP [63, 64]. Our study revealed that multiple members of these TFs are upregulated by GbMYC2, suggesting that they may be targets of GbMYC2 and play an essential role in GbMYC2-mediated TTL synthesis. Further promoter analysis revealed that the promoter of NAC3, one of the candidate target TFs of GbMYC2_4, contains a G-box motif, and eight TF promoters of the target TFs of GbMYC2_5 contain the G-box motif (Supplementary Data Table S6). Thus, these candidate TFs may play bridging roles in connecting MYC2 to TTL synthesis.

### Conclusion

**Figure 7:**
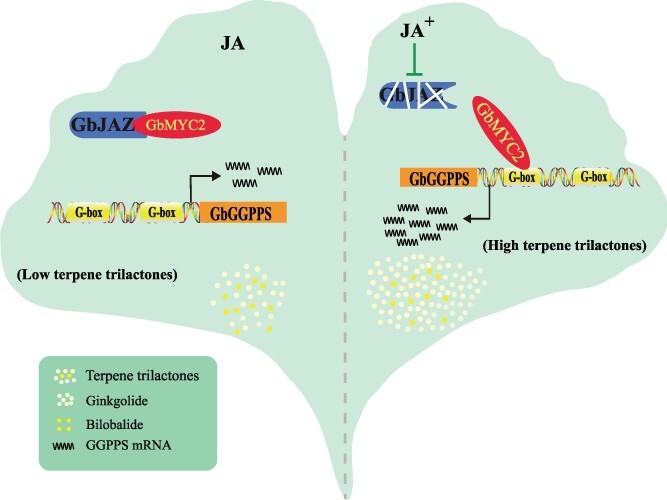
Molecular model of GbMYC2 transcription factors regulating the participation of *GbGGPPS* in TTL biosynthesis. The module, defined as GbJAZ–GbMYC2–*GbGGPPS*, relies on JA signals. When the JA content is normal or low, GbJAZs physically interacts with GbMYC2. After exogenous MeJA treatment, GbJAZs were degraded by JA signals and GbMYC2s was released. As a result of JA induction, GbMYC2_5 bound to the G-box in the promoter of *GbGGPPS* and activated its expression to promote the synthesis of ginkgolides, thereby ultimately increasing the accumulation of TTLs in the leaves. The *GbGGPPS* mRNAs are shown using wavy lines.

Based on the above findings, we identified the molecular mechanism whereby the JA-mediated GbJAZ–GbMYC2 module regulates TTL synthesis in *G. biloba*. Here, we present a working model to understand how GbJAZ–GbMYC2 regulates TTL synthesis in JA signaling (Fig. 7). At normal or low levels of JA, GbJAZs interact with GbMYC2 to inhibit GbMYC2 activity. When exogenous MeJA treatment increases the JA level in *G. biloba*, it triggers the degradation of GbJAZs in the JA signaling pathway and releases GbMYC2. As a result of JA induction, GbMYC2 binds to the G-box in the promoter of *GbGGPPS* and activates its expression to promote the synthesis of TTLs, which ultimately increases the accumulation of TTLs in the leaves. Our study reveals the molecular mechanism of MeJA-induced TTL accumulation and provides new evidence about the regulation of terpenoid synthesis through MYC2, thereby providing new approaches for breeding ginkgo with high TTL content.

## Materials and methods

### Plant materials and treatments

Ginkgo seedlings were cultured in the glass greenhouse at Yangtze University (30.35°N, 112.14°E) for 4 months. Then 4-month-old seedlings were acclimatized for 3 days after being transferred to the growth chamber (16 h light/8 h dark, 25°C), and leaves were collected after 10 mM MeJA foliar spray treatment for 2, 4, 8, 10, and 12 h, using water (containing 2% ethanol) treatment as the control. All samples were stored in a −80°C refrigerator after being snap-frozen with liquid nitrogen immediately after collection. Three biological replicates were set up for each sample. Different tissue materials were taken from 34-year-old ginkgo trees.

### RNA extraction and gene expression analysis

A TaKaRa MiniBEST Universal RNA Extraction Kit (TaKaRa, Beijing, China) was used to extract total RNA from *G. biloba* leaves. All the relative expression levels of genes in *G. biloba* were analyzed using quantitative real-time PCR (qRT–PCR). The HiScript III 1st Strand cDNA Synthesis Kit (Vazyme) was used to synthesize first-strand RNA. The qRT–PCR reactions were performed in a LineGene 9600 Plus Fluorescent Quantitative PCR System (Bioer, Hangzhou, China), and amplification was performed using ChamQ Universal SYBR qPCR Master Mix (Vazyme). The gene expression levels were normalized to the expression of *GbGAPDH* [3]. Relative expression levels were calculated with the 2^−ΔΔCt^ algorithm [2, 65]. Three biological replicates and three technical replicates were performed. Supplementary Data Table S1 lists all the primers.

### Characterization and isolation of GbMYC2s and GbJAZs

AtMYC2 was used as the query sequence to detect the *GbMYC2* gene from the genome of *G. biloba* (http://gigadb.org/dataset/100613) using BLAST, and it was confirmed as a *GbMYC2* family gene through an NCBI CD search. Primers of GbMYC2 for PCR amplification were designed and are listed in Supplementary Data Table S1. The cDNA was synthesized using a HiScript 1st Strand cDNA Synthesis Kit (Vazyme, Nanjing, China) and the full length of *GbMYC2* was amplified from the cDNA using specific primers.

Based on JAZ HMMs (PF09425 and PF06200), seven full-length coding sequences (CDSs) of *GbJAZ*s (*GbJAZ1*, *GbJAZ2*, *GbJAZ3*, *GbJAZ4*, *GbJAZ5*, *GbJAZ6*, and *GbJAZ7*) were found by HMMER 3.0. These GbJAZs were amplified from cDNA using specific primers as shown in Supplementary Data Table S1.

### Analysis of terpene trilactones using HPLC–ELSD

Total TTL content was determined using a laboratory-established high-performance liquid chromatography-evaporative light scattering detector (HPLC-ELSD) method [66]. Standards of ginkgolide J (GJ), ginkgolide B (GB), ginkgolide C (GC), ginkgolide A (GA), and bilobalide (BB) were purchased from Yuanye (Shanghai, China).

### Subcellular localization assay

The full-length ORFs of GbMYC2_4 and GbMYC2_5 were cloned into the pNC-Cam1304-SubN vector to generate SubN*-CaMV35S::EGFP-GbMYC2_4/GbMYC2_5::NOS* for the subcellular localization assay (Supplementary Data Fig. S5A). The recombinant plasmid was transformed into GV3101 by electroporation and diluted to OD600 = 1 with the resuspension solution (10 mM MES-KOH, pH = 5.6; 10 mM MgCl_2_; 100 μM AS). The above resuspension solution was mixed with an equal volume of GV3101 containing nuclear marker plasmid p2300-*CaMV35S::H2B-mCherry* (BioVector NTCC) and then injected into *N. benthamiana* leaves (five-leaf stage). The tobacco plants were incubated at 25°C (day)/23°C for 48–72 h, after which fluorescence was detected with a confocal laser scanning microscope (Leica TCS SP8, Germany).

### Acquisition of overexpressed *A. thaliana*

GbMYC2_4 and GbMYC2_5 were inserted into pNC-Cam2304-MCS35S by Nimble Cloning to construct recombinant plasmids, and then overexpression of *A. thaliana* strains was obtained using *Agrobacterium tumefaciens* GV3101 containing the recombinant plasmids by the floral dip method. Transgenic *Arabidopsis* was screened for transformants on MS medium containing 100 mg/ml kanamycin and carried out three times in succession until the *T*_3_ generation strain was obtained. Finally, PCR was used to confirm the transformation of GbMYC2 in *T*_3_ transgenic plants. *Arabidopsis thaliana* was grown in an artificial climate chamber with 16 h of light, 25°C, 8 h of darkness, 20°C, 12 000 lux of light, and 70% humidity.

### Transient expression analysis in *G. biloba* leaves

GbMYC2 was overexpressed in *G. biloba* leaves using a transient overexpression assay with reference to previous methods [66]. The empty vector (pGreenII 62-SK, SK) and expression vectors SK− gbmyc2_4 and 5 were injected into different leaves of the same ginkgo plant. Five days after injection, leaves were collected and immediately frozen in liquid nitrogen and subsequently stored in a −80°C refrigerator.

### RNA-sequencing analysis

The collected GbMYC2_4- and GbMYC2_5-overexpressing ginkgo leaves were used for transcriptome sequencing. cDNA library construction and sequencing were entrusted to Biomarker Technologies (Qingdao, China). This is briefly described as follows: cDNA libraries were sequenced using the NovaSeq 6000 platform, and after obtaining the raw data adapter sequences and low-quality sequence reads were removed to obtain clean data. The clean data were then compared with the *G. biloba* reference genome (http://gigadb.org/dataset/100613) using the Hisat2 V2.1.0 tool. Gene expression levels were characterized in fragments per kilobase of transcript (FPKM) per million fragments mapped. Differentially expressed genes (DEGs) were identified using DESeq2 with a *P*-value <0.01.

### Yeast two-hybrid assays

Y2H assays were performed using the Matchmaker Gold Yeast Two-Hybrid System (Takara, Beijing, China). The full-length ORFs of GbMYC2_4 and 5 and their truncated fragments (N, M, C-GbMYC2_4 and 5) were cloned into the EcoRI and BamHI sites of the pGBKT7 vector to form the bait plasmid, whereas GbJAZ1/2/3/4/5/6/7, N-GbJAZ2, and N-GbJAZ4 ORFs were cloned into the EcoRI and BamHI sites of the pGADT7 vector to form the prey plasmid (Supplementary Data Fig. S5D). All constructs were verified through sequencing (Sangon Biotech, Shanghai, China).

The bait and prey plasmids were transformed into yeast strains Y2HGold and Y187, respectively, using the PEG/LiAc method following the Y2HGold Chemically Competent Cell instructions (Weidi Biotechnology, Shanghai, China). The transformed Y2HGold yeast cells were cultured on a medium lacking tryptophan (SD/−T) for 3 days at 28°C, and the transformed Y187 yeast cells were cultured on a medium lacking leucine (SD/−L) for 3 days at 28°C. Yeast cells containing the corresponding plasmids were selected. Two individual transformants were randomly picked and verified through PCR and gel electrophoresis. The toxicity and autoactivation test of the bait were verified on SD/−Trp, SD/−Trp/X-α-Gal (20 μg/ml), SD/−Trp/−His/−Ade (TDO), and SD/−Trp/X-α-Gal/aureobasidin A (AbA) (200 ng/ml), and the bait was allowed to grow for 3 days at 28°C. Mating Y2HGold [pGBKT7-bait] with Y187 [pGADT7-prey] resulted in diploid cells containing both plasmids. The interactions were tested on SD/−Leu/−Trp (DDO), SD/−Leu/−Trp/−His/−Ade (QDO), QDO/X-α-Gal (QDO/X), and QDO/X-α-Gal/AbA (QDO/X/A) medium and allowed to grow for 3 days at 28°C. Additionally, pGADT7-T + pGBKT7–53 was used as a positive control, and pGADT7-T + pGBKT7-lam and pGBKT7-bait + pGADT7-empty served as negative controls. The experiments were repeated twice.

### Bimolecular fluorescence complementation assay

The full-length ORFs of *Gb*MYC2_4 and *Gb*MYC2_5 were amplified and cloned into the pNC-BiFC-Enn vector through Nimble Cloning reaction to form Enn*-CaMV35S::nEYFP-*GbMYC2_4/5*::Ter*, whereas the *GbJAZ* CDSs were cloned into the pNC-BiFC-Ecn vector through the Nimble Cloning reaction to form *pNC-CaMV35S::cEYFP-*GbJAZ1/2/3/4/6/7*::Ter* (Supplementary Data Fig. S5B). The two recombinant plasmids were transformed into GV3101 (pSoup) through electroporation and diluted to OD600 = 1 with the resuspension solution (10 mM MES-KOH, pH = 5.6; 10 mM MgCl_2_; 100 μM AS). The above *Agrobacterium* liquid was mixed with an equal volume of GV3101 containing the nuclear marker p2300-*CaMV35S::H2B-mCherry* plasmid (1:1:1) and then injected into *N. benthamiana* leaves (five-leaf stage). The tobacco was wrapped in a plastic bag for moisture retention at 25°C for 24 h in the dark, and then incubated at 25°C (day)/23°C for 36–48 h. The enhanced yellow fluorescent protein (EYFP) signal was detected with a confocal laser-scanning microscope (Leica TCS SP8, Germany).

### DAP-seq sampling and data analysis

DAP-seq was performed with reference to previous methods [30, 67], and library construction and sequencing were commissioned from Bluescape Hebei Biotech. Genomic DNA library construction was performed making use of the NGS0602- Mich TLX DNA-seq kit (Mich, Hebei, China). The CDS of GbMYC2_4 was inserted into pFN19K HaloTag T7 SP6 Flexi vector to construct a recombinant vector, and then protein expression was carried out using the TNT SP6 Coupled Wheat Germ Extract System (Promega, Madison, WI, USA), and the 50 μl reaction system was held at 37°C for 2 h to obtain the Halo-GbMYC2_4 fusi7on protein. Subsequently, the GbMYC2_4 protein was directly adsorbed with Magne HaloTag Beads (Promega) and incubated with the DNA library.

The eluted DNA was finally sequenced on the Illumina NavoSeq6000 platform, and two technical replicates were performed. A DNA library lacking the target protein was used as a negative control in the mock DAP-seq libraries. After sequencing was completed, reads were aligned to the reference *G. biloba* genome (http://gigadb.org/dataset/100613) using Bowtie 2 [68] and the peaks were processed by MEME-ChIP to obtain the conserved motifs [69]. To annotate the bound peaks we used ChIPseeker software [70].

### Yeast one-hybrid assays

The GbMYC2 target genes were screened according to the following methods. First, all the enzyme genes annotated as involved in TTL synthesis were screened from the previous transcriptomics data of our research group [2]. Then, the gene promoters containing the G-box or G-box-like motifs were retained for further analysis. Finally, nine candidate target genes containing G-box or G-box-like motifs were obtained.

Promoter fragments were inserted into the pAbAi vector to construct pAbAi_Bait. An assay was performed according to the instructions of the Matchmaker Gold Yeast One-Hybrid System (TaKaRa, Beijing, China). The pAbAi_Bait was transformed into Y1HGold and self-activation analysis was performed on selective synthetic dextrose minimal medium without uracil (SD/−U) with AbA added. Colony PCR analysis confirmed that the target sequence had been correctly integrated into the Y1HGold genome. After determining the minimum inhibitory concentration of the bait against AbA, the AD-prey vector was transformed into the Y1H[pAbAi_Bait] strain and Y1H growth analysis was performed on an SD/−U/−Leu/AbA plate containing the lowest inhibitory AbA level of the bait. The pGADT7 empty was used as a negative control, and the combination of pAbAi_53 and pGADT7_53 was used as positive control.

### Electrophoretic mobility shift assay

The ORF of GbMYC2_5 was cloned into the pCZN1 vector, and then transformed into *Escherichia coli* strain ArcticExpress (DE3) competent cells (TransGen Biotech, Beijing, China). Prokaryotic expression was performed at 16°C for 16 h with 0.2 mM isopropyl-β-d-thiogalactoside (IPTG), and then His-MYC2_5 fusion protein was obtained after purification using Ni-IDA Resin (GenScript, Nanjing, China). Biotin-labeled probes for the *GGPPS* promoter (Biotin P1, Biotin P2, Biotin P1 mutant) were synthesized by Sangon Biotech (Shanghai, China). Unlabeled probes were subjected to cold competition experiments. These primers and probes are listed in Supplementary Data Table S1. The Lightshift Chemiluminescent EMSA Kit (Thermo Scientific, Rockford, USA) was used to perform the EMSA. A single or mixed addition was performed using 0.8 μg of GbMYC2_5 and 0.1 pM probe, and incubation was performed for 25 min at 15°C. Cold probe (unlabeled competitor) additions of 20-fold and 50-fold were carried out for competitive EMSA experiments.

### Dual-luciferase assay

As previously described [71], the transcriptional activation activity between GbMYC2_5 and the target promoter were measured using a dual-luciferase assay. The promoters GbGGPPS_Pro1 and GbGGPPS_Pro2 (101 bp) were inserted into the pGreenII 0800-LUC vector, while the encoding sequence of GbMYC2_5 was inserted into pGreenII 62-SK to generate the effector (Fig. 6D, Supplementary Data Fig. S5E). Reporter and effector structures were transformed by electroporation into *Agrobacterium* GV3101 (pSoup) for penetration into 4-week-old tobacco leaves. After mixing with SK:Luc = 10:1, the tobacco leaves were injected with a needle-free syringe. *Nicotiana benthamiana* plants were grown under conditions of 24°C during the day/20°C at night with a photoperiod of light/dark 16/8 h. After 48 h, firefly luciferase (LUC) and *Renilla* (REN) luciferase activities were measured in a microplate reader (Sense 425–301, Hidex) using a Dual Luciferase Reporter Assay Kit (Vazyme). The assays were performed with four biological replicates.

### Statistical analysis

The figures were generated using OriginPro 9.0 software (OriginLab, USA). Error bars in the figures indicate the standard deviation of biological replicates. Statistical analyses were completed utilizing SPSS 22.0 (SPSS, IBM, USA) and GraphPad Prism 8.0. Analysis of variance was performed using Fisher's LSD test and the *t*-test. Two-factor ANOVA was used to handle significant comparisons between multiple groups.

### Accession numbers

NtMYC2: NP_001313001.1, *N. tabacum*; AaMYC2, AKO62850.1, *A. annua*; AtMYC2, AT1G32640.1, *A. thaliana*; ZmMYC2: ON643092, *Z. mays*; AtMYC3, AT5G46760.1, *A. thaliana*; AtMYC4: AT4G17880.1, *A. thaliana*; TmMYC2, AHL44338.1, *T. mairei*; TcMYC2a: ATY38591.1, *T. chinensis*; AtMYC1: AT4G00480.1; AtMYC-2: AT1G63650.1; AtMYC6: AT5G41315.1.

## Supplementary Material

Web_Material_uhae228

## Data Availability

All relevant data can be found within the manuscript and its supporting materials. The RNA-seq data have been deposited in the China National GeneBank DataBase: CNP0004970. DAP-seq data have been deposited in the China National GeneBank DataBase: CNP0004964.
