# Supplementary material for: The transcription factor MYC2 positively regulates terpene trilactone biosynthesis through activating GbGGPPS expression in Ginkgo biloba
Source: Hortic Res. 2024 Aug 9;11(10):uhae228. doi: 10.1093/hr/uhae228 (PMC11480656; doi:10.1093/hr/uhae228)
Supplement: Web_Material_uhae228 [file web_material_uhae228.zip › Figure S1-S5.pdf]

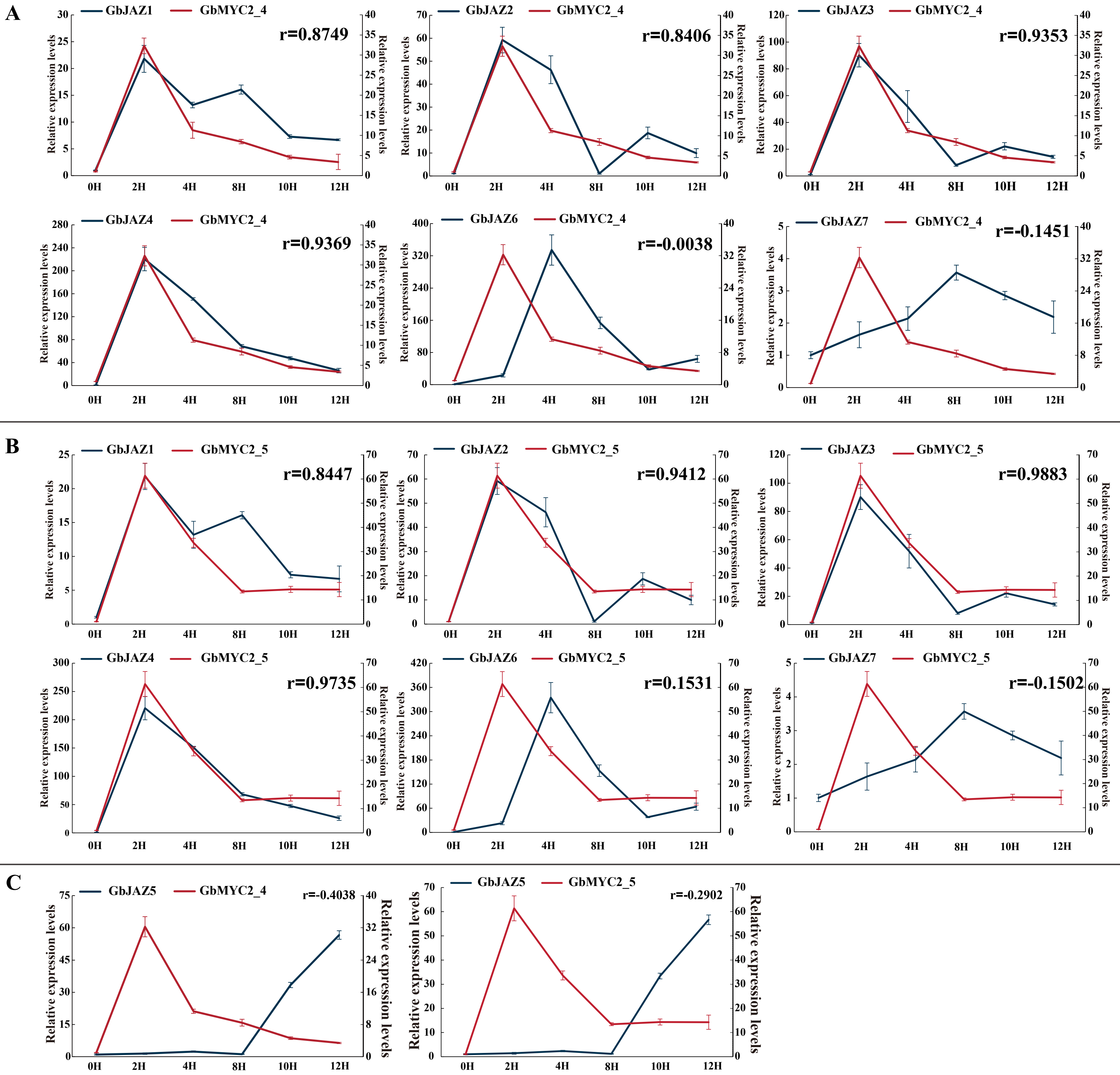

A
GbJAZ1
GbMYC2_4
r=0.8749
GbJAZ2
GbMYC2_4
r=0.8406
GbJAZ3
GbMYC2_4
r=0.9353
GbJAZ4
GbMYC2_4
r=0.9369
GbJAZ6
GbMYC2_4
r=-0.0038
GbJAZ7
GbMYC2_4
r=-0.1451
B
GbJAZ1
GbMYC2_5
r=0.8447
GbJAZ2
GbMYC2_5
r=0.9412
GbJAZ3
GbMYC2_5
r=0.9883
GbJAZ4
GbMYC2_5
r=0.9735
GbJAZ6
GbMYC2_5
r=0.1531
GbJAZ7
GbMYC2_5
r=-0.1502
C
GbJAZ5
GbMYC2_4
r=-0.4038
GbJAZ5
GbMYC2_5
r=-0.2902
Relative expression levels
0H
2H
4H
8H
10H
12H

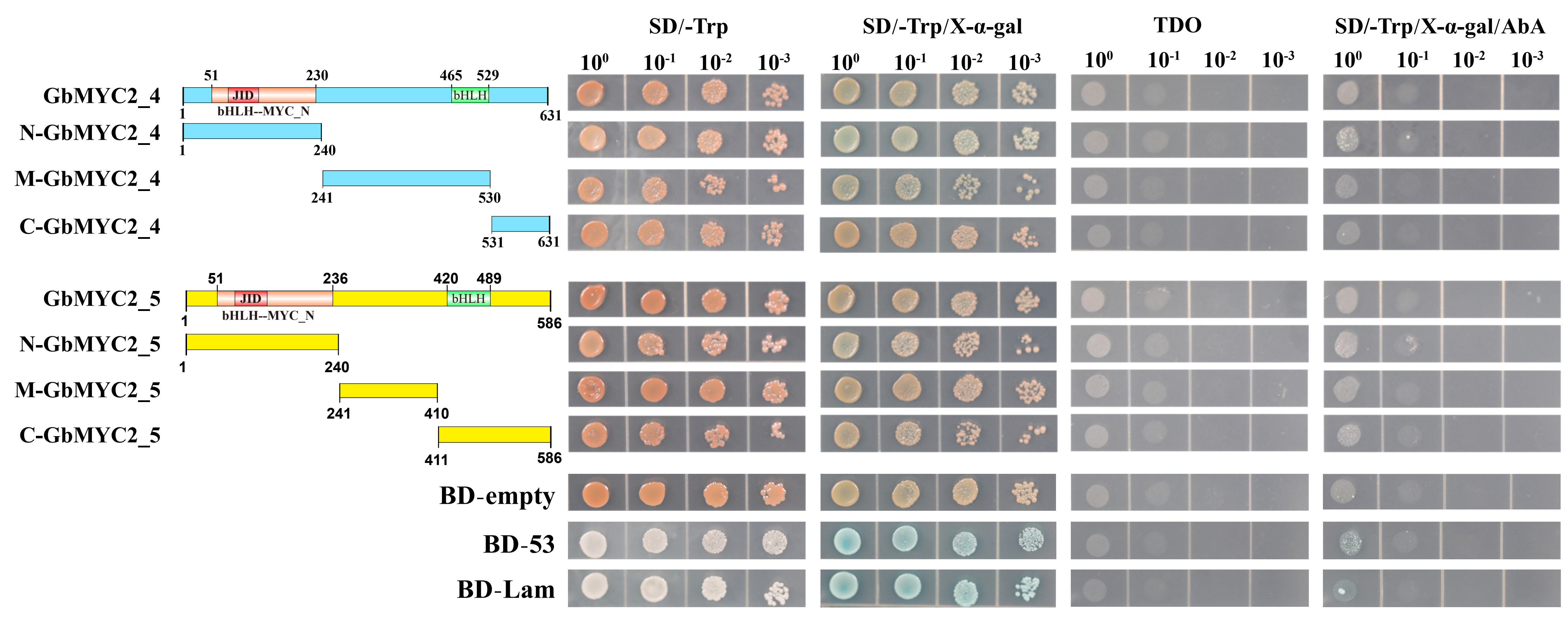

SD/-Trp
SD/-Trp/X-α-gal
TDO
SD/-Trp/X-α-gal/AbA
10^0 10^{-1} 10^{-2} 10^{-3}
GbMYC2_4
51 230 465 529
JID
bHLH
bHLH--MYC_N
1 631
N-GbMYC2_4
1 240
M-GbMYC2_4
241 530
C-GbMYC2_4
531 631
GbMYC2_5
51 236 420 489
JID
bHLH
bHLH--MYC_N
1 586
N-GbMYC2_5
1 240
M-GbMYC2_5
241 410
C-GbMYC2_5
411 586
BD-empty
BD-53
BD-Lam

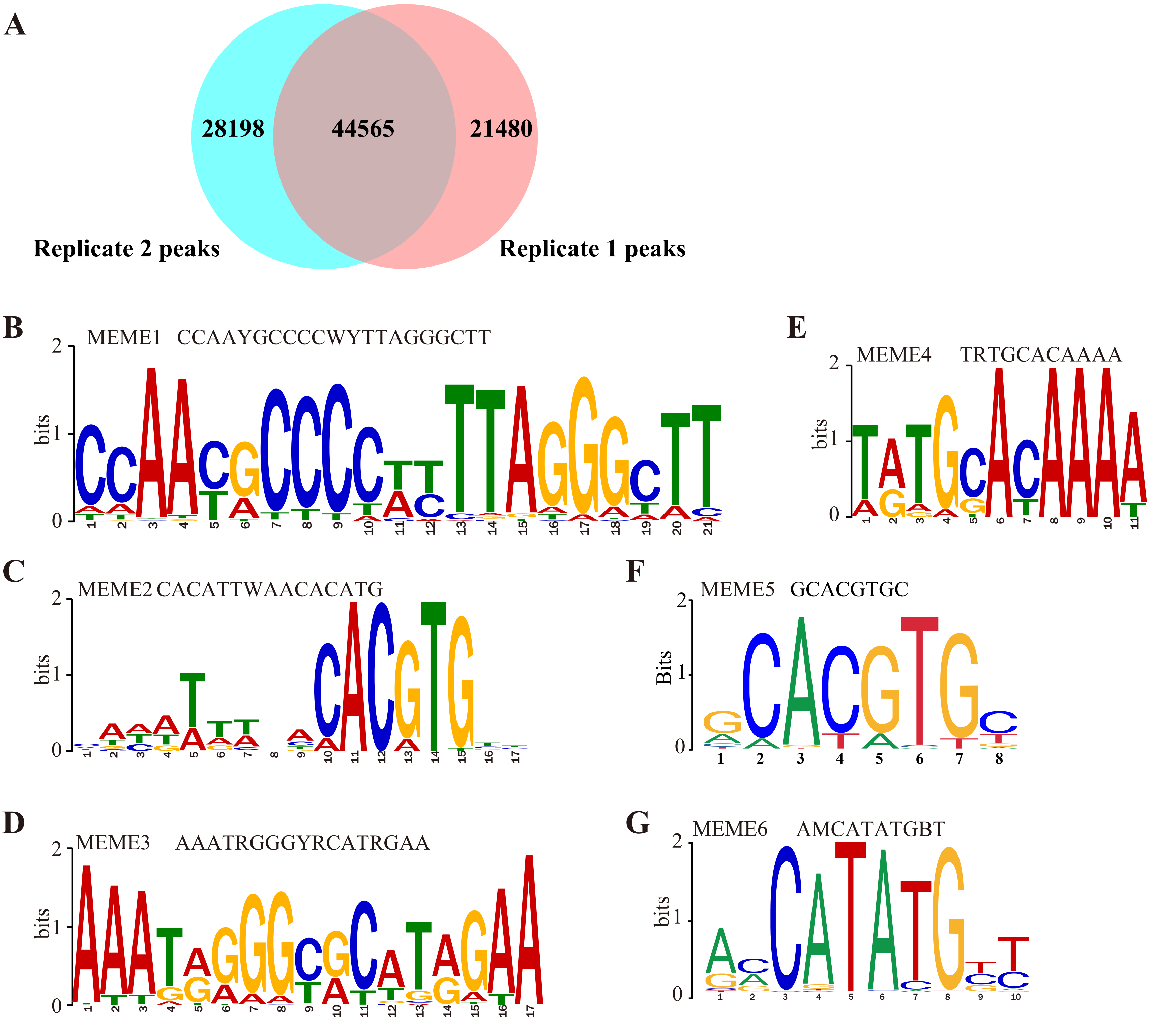
A
28198
44565
21480
Replicate 2 peaks
Replicate 1 peaks
B
MEME1 CCAAYGCCCCWYTTAGGGCTT
bits
C
MEME2 CACATTWAACACATG
bits
D
MEME3 AAATRGGGYRCATRGAA
bits
E
MEME4 TRTGCACAAAA
bits
F
MEME5 GCACGTGC
Bits
G
MEME6 AMCATATGBT
bits

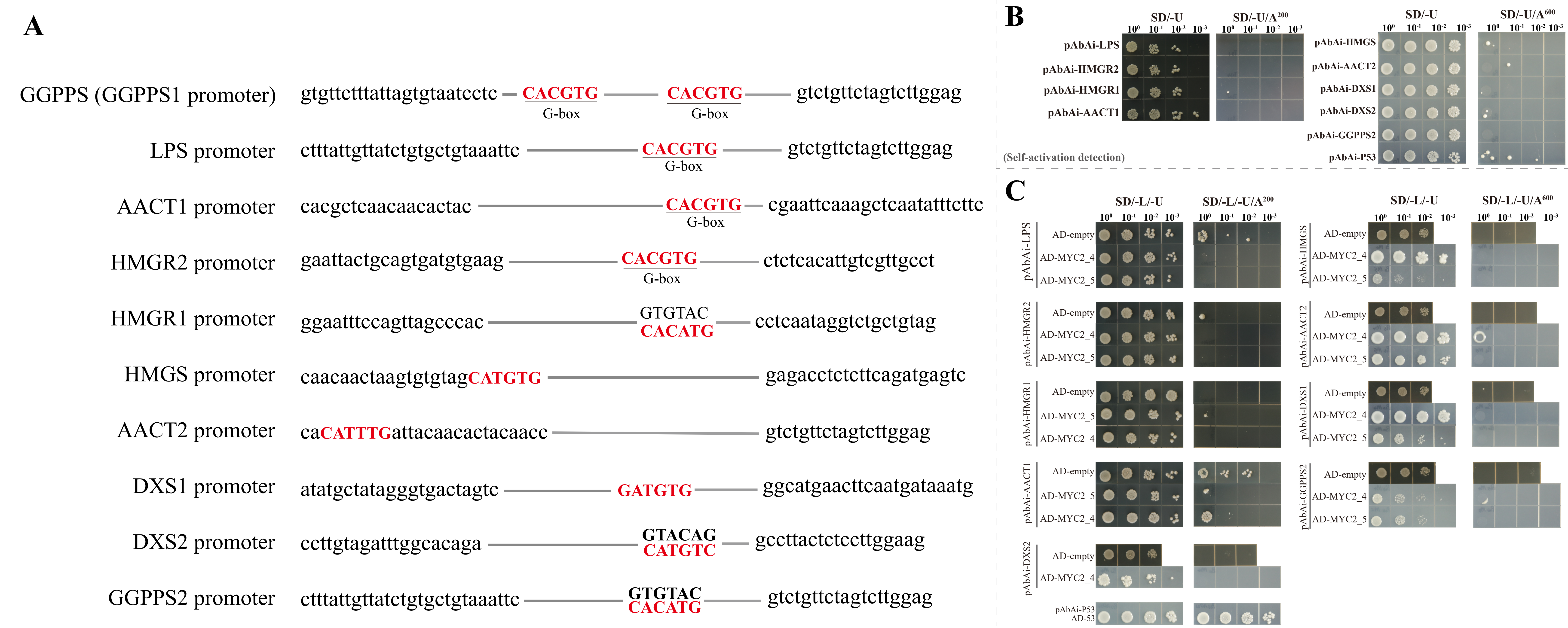
A
GGPPS (GGPPS1 promoter)
gtgttctttattagtgtaatcctc
CACGTG
G-box
CACGTG
G-box
gtctgttctagtcttggag
LPS promoter
ctttattgttatctgtgctgtaaattc
CACGTG
G-box
gtctgttctagtcttggag
AACT1 promoter
cacgctcaacaacactac
CACGTG
G-box
cgaattcaaagctcaatatttcttc
HMGR2 promoter
gaattactgcagtgatgtgaag
CACGTG
G-box
ctctcacattgtcgttgcct
HMGR1 promoter
ggaatttccagttagcccac
GTGTAC
CACATG
cctcaataggtctgctgtag
HMGS promoter
caacaactaagtgtgtagCATGTG
gagacctctcttcagatgagtc
AACT2 promoter
caCATTTGattacaacactacaacc
gtctgttctagtcttggag
DXS1 promoter
atatgctatagggtgactagtc
GATGTG
ggcatgaacttcaatgataaatg
DXS2 promoter
ccttgtagatttggcacaga
GTACAG
CATGTC
gccttactctccttggaag
GGPPS2 promoter
ctttattgttatctgtgctgtaaattc
GTGTAC
CACATG
gtctgttctagtcttggag
B
SD/-U
SD/-U/A200
pAbAi-LPS
pAbAi-HMGR2
pAbAi-HMGR1
pAbAi-AACT1
pAbAi-HMGS
pAbAi-AACT2
pAbAi-DXS1
pAbAi-DXS2
pAbAi-GGPPS2
pAbAi-P53
SD/-U/A600
(Self-activation detection)
C
SD/-L/-U
SD/-L/-U/A200
SD/-L/-U/A600
pAbAi-LPS
pAbAi-HMGR2
pAbAi-HMGR1
pAbAi-AACT1
pAbAi-DXS2
pAbAi-HMGS
pAbAi-AACT2
pAbAi-DXS1
pAbAi-GGPPS2
AD-empty
AD-MYC2_4
AD-MYC2_5
pAbAi-P53
AD-53

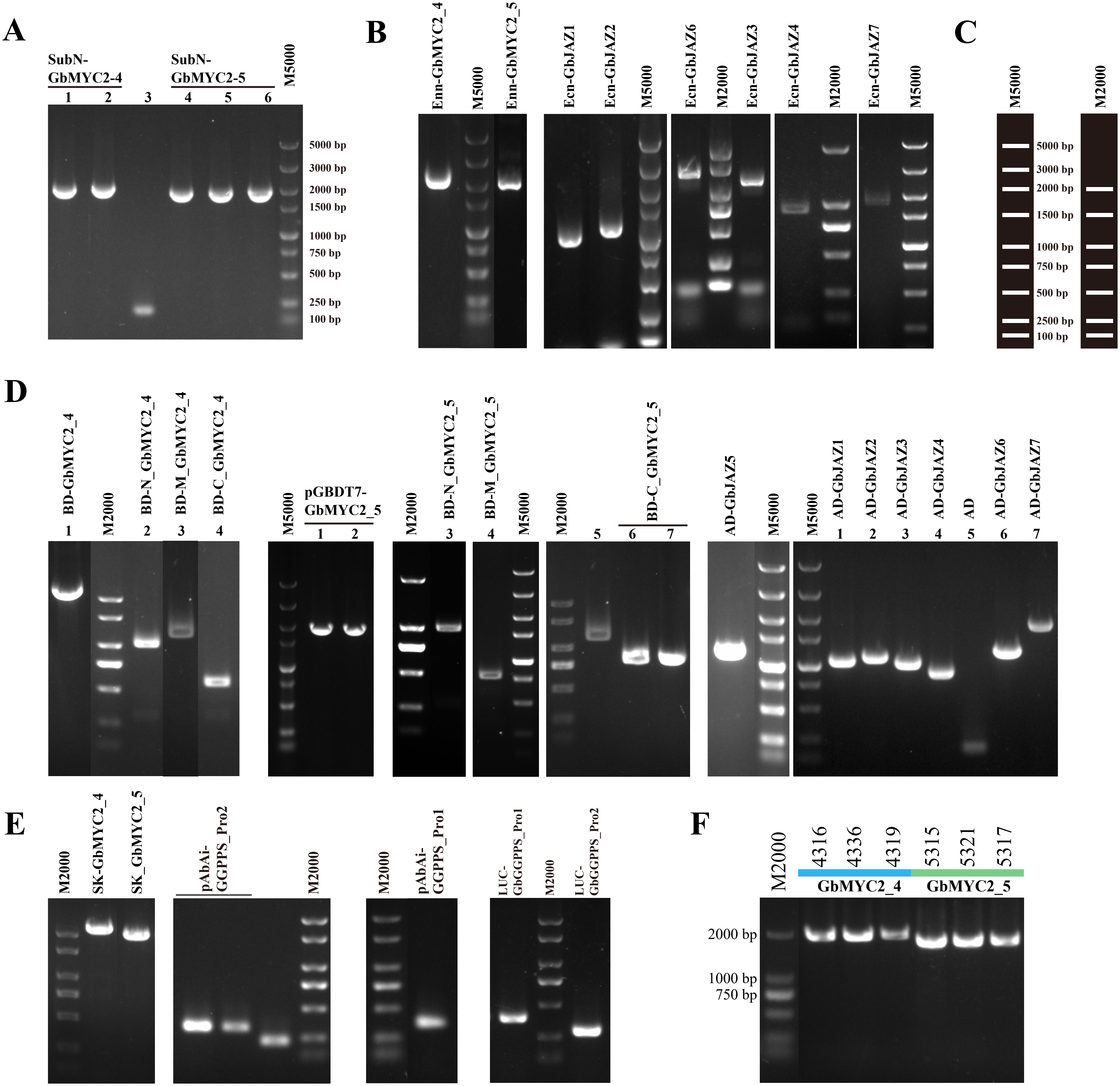
A
SubN-GbMYC2-4
SubN-GbMYC2-5
1 2 3 4 5 6
M5000
5000 bp
3000 bp
2000 bp
1500 bp
1000 bp
750 bp
500 bp
250 bp
100 bp
B
Enn-GbMYC2_4
M5000
Enn-GbMYC2_5
Ecn-GbJAZ1
Ecn-GbJAZ2
M5000
Ecn-GbJAZ6
M2000
Ecn-GbJAZ3
Ecn-GbJAZ4
M2000
Ecn-GbJAZ7
M5000
C
M5000
M2000
5000 bp
3000 bp
2000 bp
1500 bp
1000 bp
750 bp
500 bp
2500 bp
100 bp
D
BD-GbMYC2_4
M2000
BD-N_GbMYC2_4
BD-M_GbMYC2_4
BD-C_GbMYC2_4
1 2 3 4
M5000
pGBDT7-GbMYC2_5
1 2
M2000
BD-N_GbMYC2_5
BD-M_GbMYC2_5
M5000
3 4
M2000
BD-C_GbMYC2_5
5 6 7
AD-GbJAZ5
M5000
M5000
AD-GbJAZ1
AD-GbJAZ2
AD-GbJAZ3
AD-GbJAZ4
AD
AD-GbJAZ6
AD-GbJAZ7
1 2 3 4 5 6 7
E
M2000
SK-GbMYC2_4
SK_GbMYC2_5
pAbAi-GGPPS_Pro2
M2000
M2000
pAbAi-GGPPS_Pro1
LUC-GbGGPPS_Pro1
M2000
LUC-GbGGPPS_Pro2
F
M2000
4316 4336 4319 5315 5321 5317
GbMYC2_4
GbMYC2_5
2000 bp
1000 bp
750 bp
