# Supplementary material for: The transcription factor MYC2 positively regulates terpene trilactone biosynthesis through activating GbGGPPS expression in Ginkgo biloba
Source: Hortic Res. 2024 Aug 9;11(10):uhae228. doi: 10.1093/hr/uhae228 (PMC11480656; doi:10.1093/hr/uhae228)
Supplement: Web_Material_uhae228 [file web_material_uhae228.zip › Supplemental materials (FigureS1-S5).docx]

**The transcription factor MYC2 positively regulates terpene trilactone biosynthesis through activating *GbGGPPS* expression in *Ginkgo biloba***

Jiarui Zheng^†^, Yongling Liao^†^, Jiabao Ye^†^, Feng Xu*, Weiwei Zhang, Xian Zhou, Lina Wang, Xiao He, Zhengyan Cao, Yuwei Yi, Yansheng Xue, Qiangwen Chen, Jiaxing Sun

College of Horticulture and Gardening, Yangtze University, Jingzhou, 434025, China

†These authors contributed equally to this work.

*Corresponding author: Feng Xu, E-mail: xufeng@yangtzeu.edu.cn (ORCID: 0000-0003-3212-6284)

**Email addresses of all authors:**

Jiarui Zheng: 2021730075@yangtzeu.edu.cn; Yongling Liao: liaoyongling@yangtzeu.edu.cn; Jiabao Ye: yejiabao@yangtzeu.edu.cn; Feng Xu: xufeng@yangtzeu.edu.cn; Weiwei Zhang: wwzhang@yangtzeu.edu.cn; Xian Zhou: xian_zhou.st@yangtzeu.edu.cn; Lina Wang: 521067@yangtzeu.edu.cn; Xiao He: 202071709@yangtzeu.edu.cn; Zhengyan Cao: 202071705@yangtzeu.edu.cn; Yuwei Yi: 2022710850@yangtzeu.edu.cn; Yansheng Xue: 201971432@yangtzeu.edu.cn; Qiangwen Chen: 201973054@yangtzeu.edu.cn; Jiaxing Sun: 2022710863@yangtzeu.edu.cn.

**Running head:** GbMYC2-GbGGPPS module regulates TTL biosynthesis


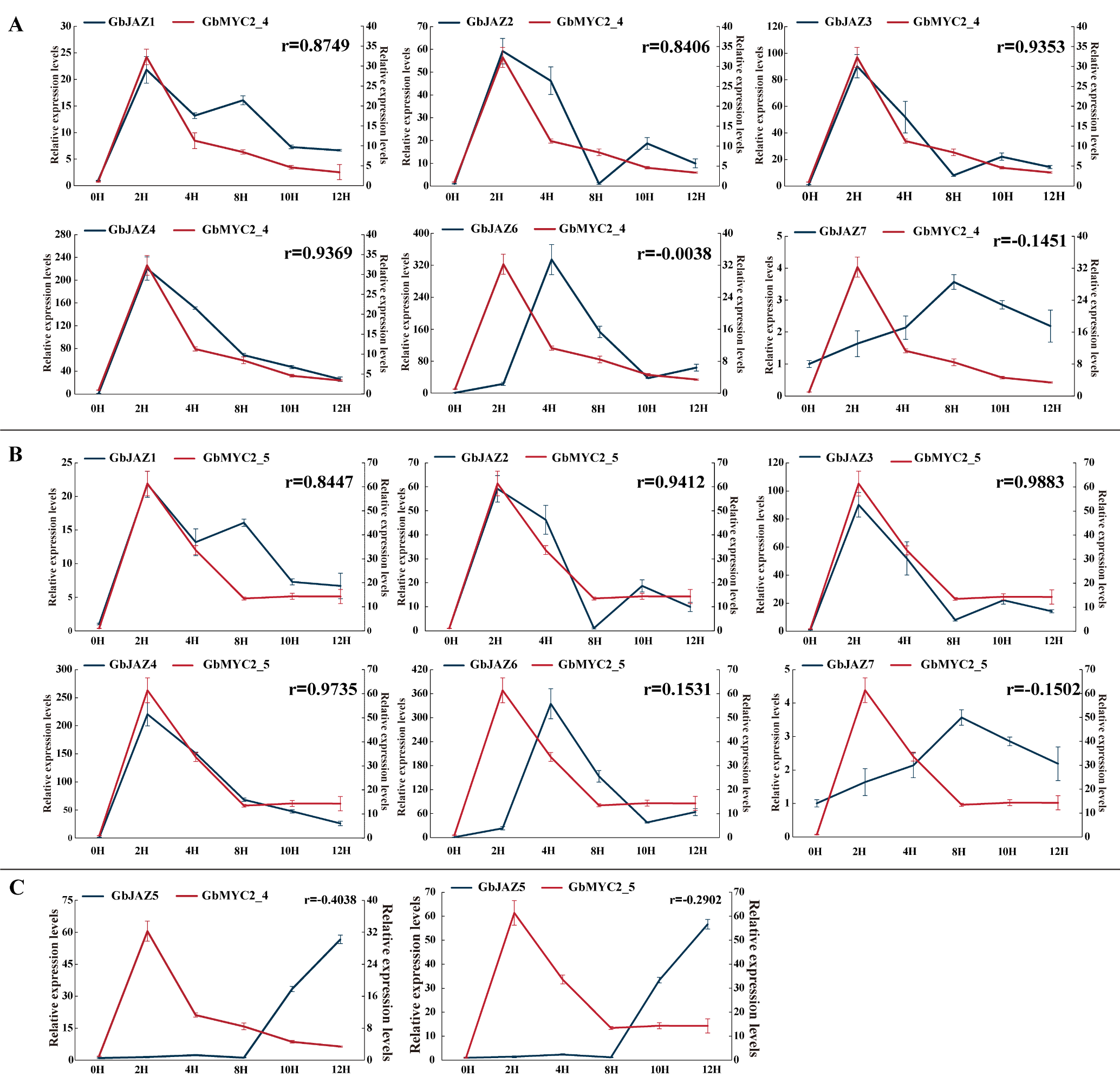


**Fig. S1** Correlation analysis between the expression levels of GbJAZ and GbMYC2. **(A)** Correlation between six GbJAZs and GbMYC2_4. **(B)** Correlation between six GbJAZs and GbMYC2_5. **(C)** Correlation of GbJAZ5 with GbMYC2_4 and GbMYC2_5. They show the lowest correlation with each other.


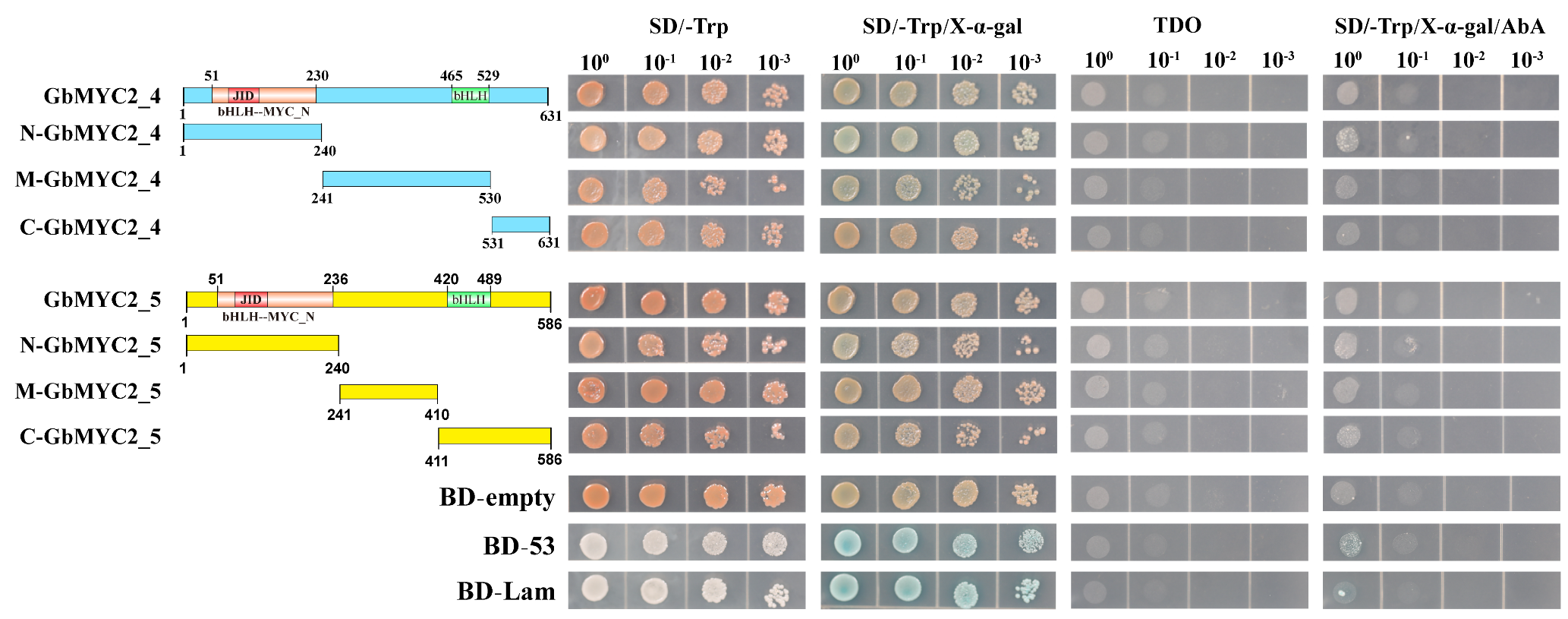


**Fig. S2** Self-activation analysis of GbMYC2s in yeast cells. Each GbMYC2 was fused with the DNA-binding domain (BD) in pGBKT7. Validation of the self-activation of GbMYC2-4/5 and different fragments in yeast cells. The results of the self-activation validation of the full-length and truncated fragments of GbMYC2_4 and GbMYC2_5 are shown in detail. The number represents the amino acid number. The growth situation of Y2H yeast containing the recombinant vector in SD/-Trp, SD/-Trp/X-α-gal, SD/-Trp/-His/-Ade (TDO), and SD/-Trp/X-α-gal/AbA media.


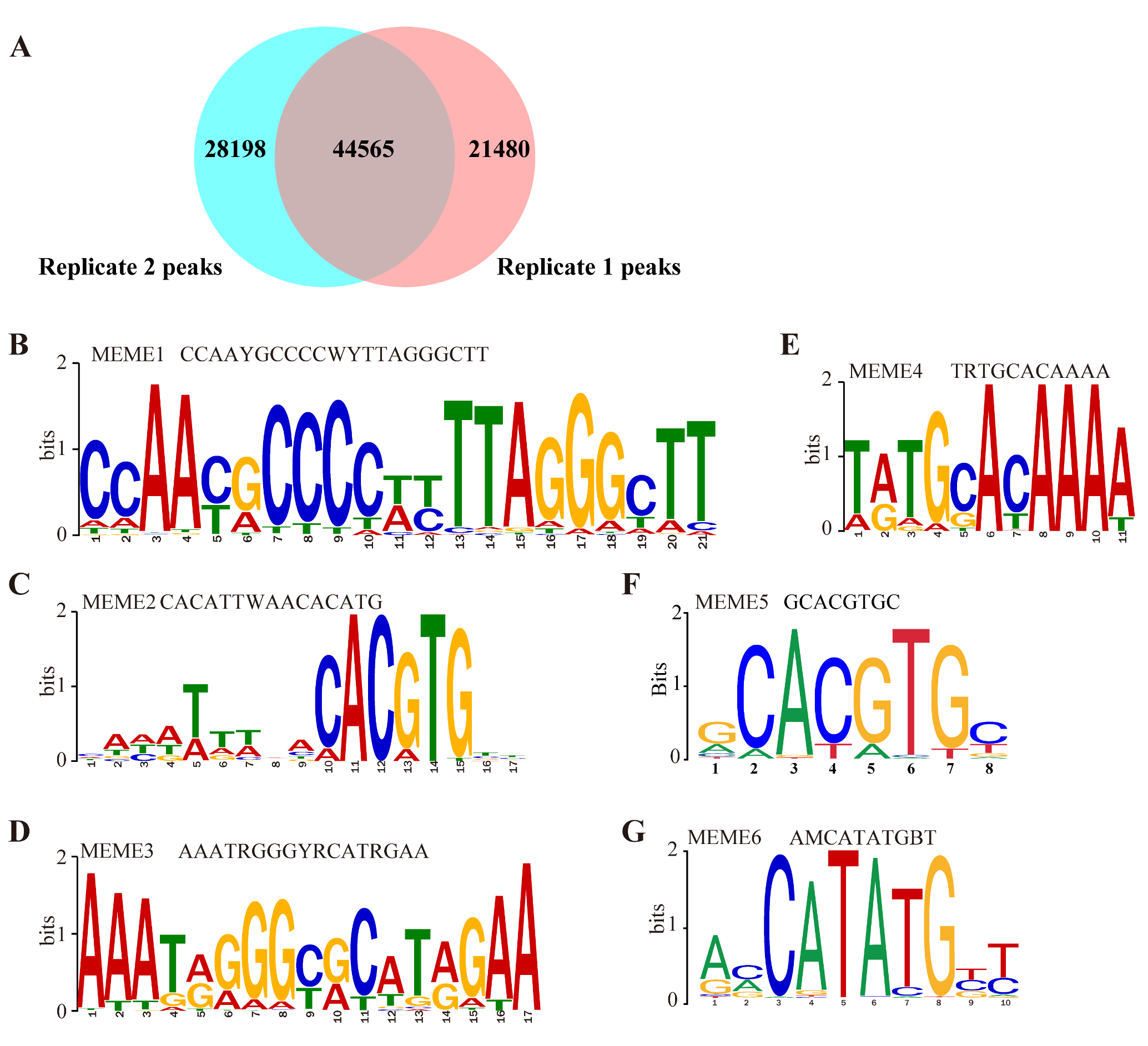


**Fig. S3** Genome-wide identification of GbMYC2_4 target by DAP-seq. **(A)** Venn diagram depicting number of MYC2 binding peaks in two biological replicates of DAP-seq analysis. **(B-G)** The top six consensus GbMYC2_4-binding DNA motifs identified by MEME.


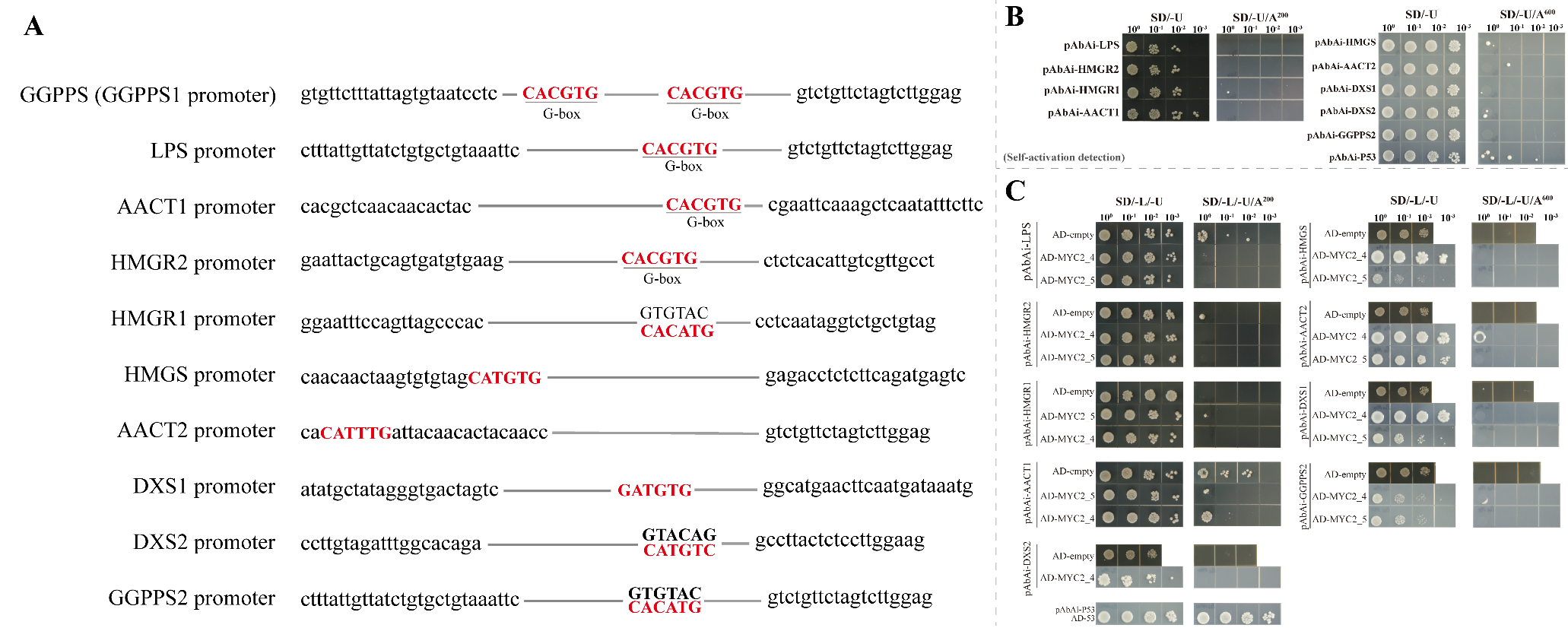


**Fig. S4** GbMYC2_4 and GbMYC2_5 binding activity to promoters of candidate genes containing G-box motifs was tested by yeast one-hybrid assay. **(A)** Position of G-box and G-box like in the promoters of candidate genes. **(B-C)** Analysis of self-activation in Y1H cells after recombination of the *LPS*, *AACT1*, *AACT2*, *HMGR1*, *HMGR2*, *HMGS*, *DXS1*, *DXS2*, *GGPPS2* gene promoters with the pAbAi vector. Aureobasidin A (AbA) was used as a reporter.


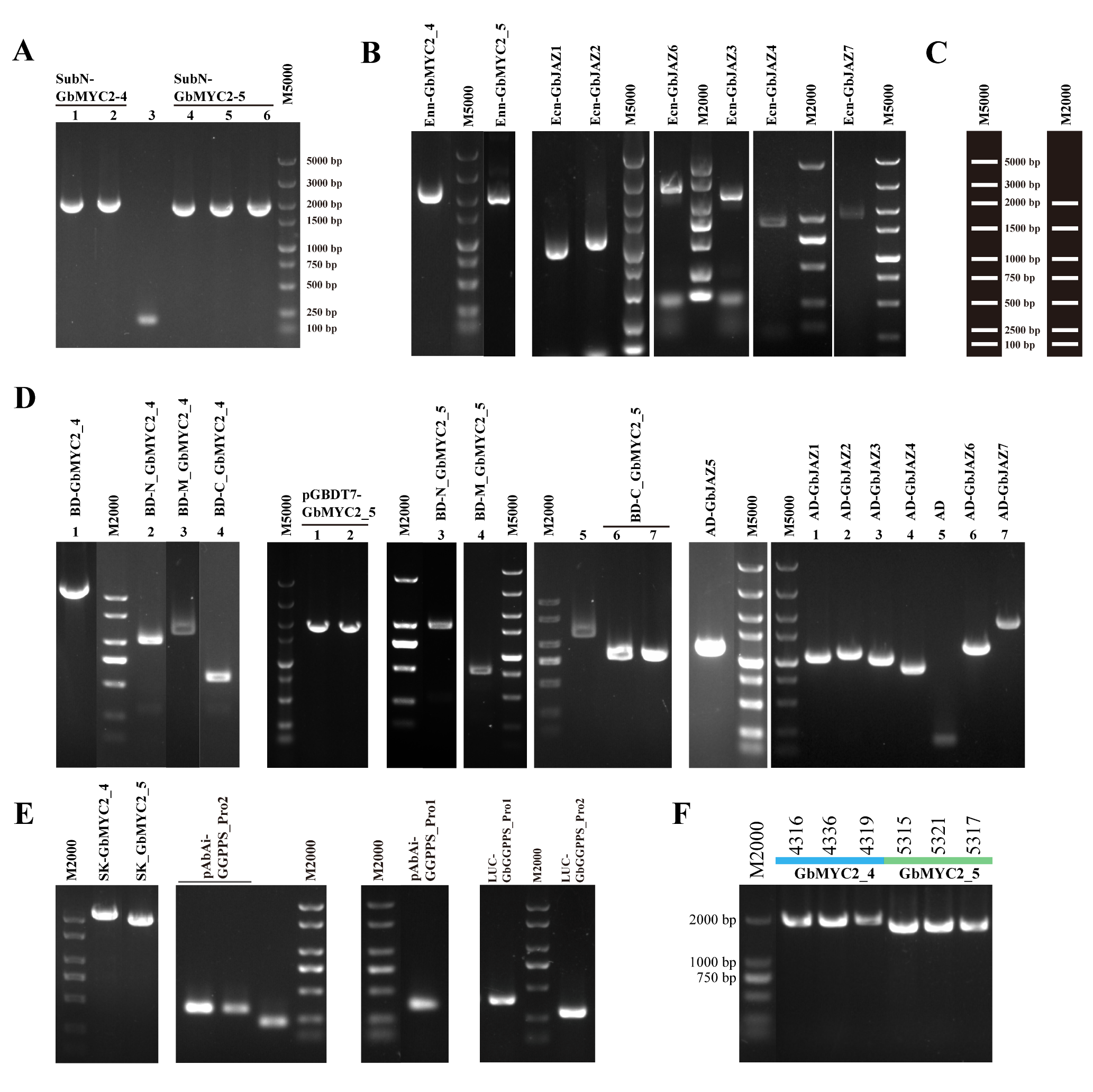


**Fig. S5** Genes cloning and PCR products of recombinant plasmids were verified by gel electrophoresis in this study. Electrophoretic detection of target fragment insertion vectors for subcellular localization **(A)**, BiFC **(B)**, Y2H **(D)**, dual-luciferase **(E)** assays. **(C)** 2000 bp and 5000 bp DNA marker were used as DNA molecular weight standards. **(F)** PCR results of DNA from transgenic *Arabidopsis thaliana* were used to detect the transfer of the GbMYC2.
